# Large scale mitochondrial sequencing in Mexican Americans suggests a reappraisal of Native American origins

**DOI:** 10.1186/1471-2148-11-293

**Published:** 2011-10-07

**Authors:** Satish Kumar, Claire Bellis, Mark Zlojutro, Phillip E Melton, John Blangero, Joanne E Curran

**Affiliations:** 1 Department of Genetics, Texas Biomedical Research Institute, San Antonio, Texas 78227, USA

## Abstract

**Background:**

The Asian origin of Native Americans is largely accepted. However uncertainties persist regarding the source population(s) within Asia, the divergence and arrival time(s) of the founder groups, the number of expansion events, and migration routes into the New World. mtDNA data, presented over the past two decades, have been used to suggest a single-migration model for which the Beringian land mass plays an important role.

**Results:**

In our analysis of 568 mitochondrial genomes, the coalescent age estimates of shared roots between Native American and Siberian-Asian lineages, calculated using two different mutation rates, are A4 (27.5 ± 6.8 kya/22.7 ± 7.4 kya), C1 (21.4 ± 2.7 kya/16.4 ± 1.5 kya), C4 (21.0 ± 4.6 kya/20.0 ± 6.4 kya), and D4e1 (24.1 ± 9.0 kya/17.9 ± 10.0 kya). The coalescent age estimates of pan-American haplogroups calculated using the same two mutation rates (A2:19.5 ± 1.3 kya/16.1 ± 1.5 kya, B2:20.8 ± 2.0 kya/18.1 ± 2.4 kya, C1:21.4 ± 2.7 kya/16.4 ± 1.5 kya and D1:17.2 ± 2.0 kya/14.9 ± 2.2 kya) and estimates of population expansions within America (~21-16 kya), support the pre-Clovis occupation of the New World. The phylogeography of sublineages within American haplogroups A2, B2, D1 and the C1b, C1c andC1d subhaplogroups of C1 are complex and largely specific to geographical North, Central and South America. However some sub-branches (B2b, C1b, C1c, C1d and D1f) already existed in American founder haplogroups before expansion into the America.

**Conclusions:**

Our results suggest that Native American founders diverged from their Siberian-Asian progenitors sometime during the last glacial maximum (LGM) and expanded into America soon after the LGM peak (~20-16 kya). The phylogeography of haplogroup C1 suggest that this American founder haplogroup differentiated in Siberia-Asia. The situation is less clear for haplogroup B2, however haplogroups A2 and D1 may have differentiated soon after the Native American founders divergence. A moderate population bottle neck in American founder populations just before the expansion most plausibly resulted in few founder types in America. The similar estimates of the diversity indices and Bayesian skyline analysis in North America, Central America and South America suggest almost simultaneous (~ 2.0 ky from South to North America) colonization of these geographical regions with rapid population expansion differentiating into more or less regional branches across the pan-American haplogroups.

## Background

Archeological, anatomical, linguistic, and genetic evidence have collectively shown that the original human inhabitants of the Western Hemisphere most likely arrived from Asia across an exposed land mass between northeastern Siberia and Alaska, referred to as Beringia, during the last glacial maximum (LGM) approximately 23 to 19 thousand years ago (kya) [1-6]. However, despite the general consensus of an Asian origin for Native Americans, a number of uncertainties persist regarding the dynamic nature of the peopling of the Americas, including the ancestral source population(s) within Asia, the arrival time(s) of the founder groups, the number of expansion events, and the specific migration routes into the New World [2,7-15].

Mitochondrial DNA (mtDNA) data presented over the past two decades have shown that Native American populations exhibit, almost exclusively, five mtDNA haplogroups (A-D and X) [6] classified in the autochthonous American lineages A2, B2, C1, D1, and X2a [16]. Haplogroups A - D are found throughout the New World and are frequent in Asia, supporting a northeastern Asian origin of these lineages [17,18]. The pan-American distribution of haplogroups A2, B2, C1 and D1, along with their similar levels of diversity and estimates for coalescence time, has been used to support a single-migration model [19-22]. Nested within this model, the Beringian land mass has been suggested to play an important role as a region where ancestral populations crossed and perhaps settled prior to their eventual expansion into the Americas, leading to the diversification of the New World founding lineages [23-26]. The evolutionary history of the fifth founder lineage, X2a, remains elusive and highly debated, with some scholars arguing for an additional migration event into the New World to account for the X lineage, one that was independent from the founder populations responsible for introducing the pan-American haplogroups [27,28].

The "Beringian incubation model" (BIM) [23] and its variants [21,25,26] emphasize that the Native American founder population reached greater Beringia by 30 kya, marked by the earliest evidence of human habitation in northeastern Siberia Yana Rhinoceros Horn Site [29]. According to these models, the founder populations remained in Beringian LGM refugium for about 5-15 kya, ecologically isolated to the west and physically isolated to the east by the glaciers that are believed to have effectively blocked the way to America until near the end of the LGM [24]. During this time, mtDNA lineages of American founders are likely to have differentiated from their Asian precursors. As the Laurentide and Cordilleran ice sheets retreated, the paused populations rapidly colonized the double continent around 14-16 kya, resulting in largely autochthonous patterns of variation within the continental founder haplogroups [23,25,26].

However, the timing of this peopling scenario has been questioned due to the existence of pre-Clovis archaeological sites, such as the Monte Verde site in southern South America that dates human habitation by at least 14.5 kya [30], as well as demographic models based on mtDNA variation that indicate pronounced growth of Native American founder populations starting at ~19 to18 kya [24], thus marking the effective colonization of the New World prior to the opening of the ice free corridors in North America. To accommodate these dates, an alternate early coastal route has been suggested for the initial colonization of the Americas, which would have been largely ice free at ~19 kya [24,31-33].

In northeastern Siberia, the putative origin of the Native American founder populations, there is archaeological evidence for human presence as early as 30 kya [29]. The estimated dates of the Beringian 'pause', (ranging from 5 to 15 thousand years) [23,24,26] and its potential role as a time when American founders diverged from their Siberian-Asian/Asian ancestors represent interesting avenues for microevolutionary reconstruction. Furthermore, the proposition for a Beringian pause [23,24,26] is largely based on the assumption that the American founder haplogroups differentiated during the Beringian pause/isolation. Several related issues remain to be addressed. First, despite the fact that much of interior Beringia remains in contemporary Alaska and northeast Siberia, no archaeological evidence of this population in residence has been found [34]. Second, a resident, presumably stable and possibly growing, population in Beringia seems an unlikely candidate for reduced genetic diversity in American founders as a result of founder effect. Third, while Chukchi and Siberian Eskimos share mtDNA lineages with Alaskan Eskimos, Aleuts and other Native North Americans [9,35], this sharing falls almost exclusively under haplogroups A2 and D2. The restricted genetic variation observed among these populations (only haplogroups A and D) seems inconsistent as a source area for the more extensive genetic variation observed in the rest of the Americas. Furthermore, the presence of haplogroup D2 in the Beringians is attributed to a second expansion from southern Siberia [36-39] and A2 is limited in diversity in Beringia [35,38].

In this study, we analyzed 568 mitochondrial genomes (of which 215 are newly sequenced from a Mexican American population) belonging to four pan-Native American (A2, B2, C1 and D1) lineages and A2a, A2b, A4b, C1a, C4 and D4e1 sister clades from Siberia-Asia, to better understand the underlying processes of the diversification of the Native American founders from their Asian counterparts and their expansion into the New World.

## Results

### Maternal legacy of Mexican Americans

Mexicans are, by and large, descendants of Native American and European (Spanish) ancestors [40]. Historical accounts also document African slavery in Mexico during the 16th-18th centuries [41-43], another source of admixture in the Mexican population. The admixture estimates compiled by Lisker et al. [44] using data derived from classical genetic systems reported in previous studies in Mexico [45-52] identified African and/or European genetic variation in all Mexican regions and groups analyzed. For mtDNA variation, some studies have measured Native American, European and African contributions to Mexican and Mexican American populations, revealing 85 to 90% of mtDNA lineages are of Native American origin [53,54], with the remainder having European (5-7%) or African ancestry (3-5%) [54]. Thus the observed frequency of Native American mtDNA in Mexican/Mexican Americans is higher than was expected on the basis of autosomal estimates of Native American admixture for these populations i.e. ~ 30-46% [53,55]. The difference is indicative of directional mating involving preferentially immigrant men and Native American women. This type of genetic asymmetry has been observed in other populations, including Brazilian individuals of African ancestry, as the analysis of sex specific and autosomal markers has revealed evidence for substantial European admixture that was mediated mostly through men [56]. In our 384 completely sequenced Mexican American mitochondrial genomes, 12 (3.1%) are of African ancestry belonging to haplogroups L0a1a'3', L2a1, L3b, L3d and U6a7; 52 (13.6%) belong to European haplogroups HV, JT, U1, U4, U5; and K and the majority (320, 83.3%) are of Native American ancestry, which is very similar to previous reports [53,54].

After removing the related individuals from our maternal lines and those of European or African descent, 215 newly sequenced unrelated mtDNA genomes of Native American ancestry were identified for founder haplogroups A2, B2, C1, D1 and D4e1c (new sister lineage of D2 found in Mexican Americans). These complete mtDNA sequences were analyzed together with 353 previously published sequences belonging to the four primary Native American founder haplogroups A2, B2, C1 and D1 and their Asian sister clades (A4, C4 and D4e; see additional file 1 for details).

### Phylogenetic reconstructions and coalescent age estimates

**Figure 1:**
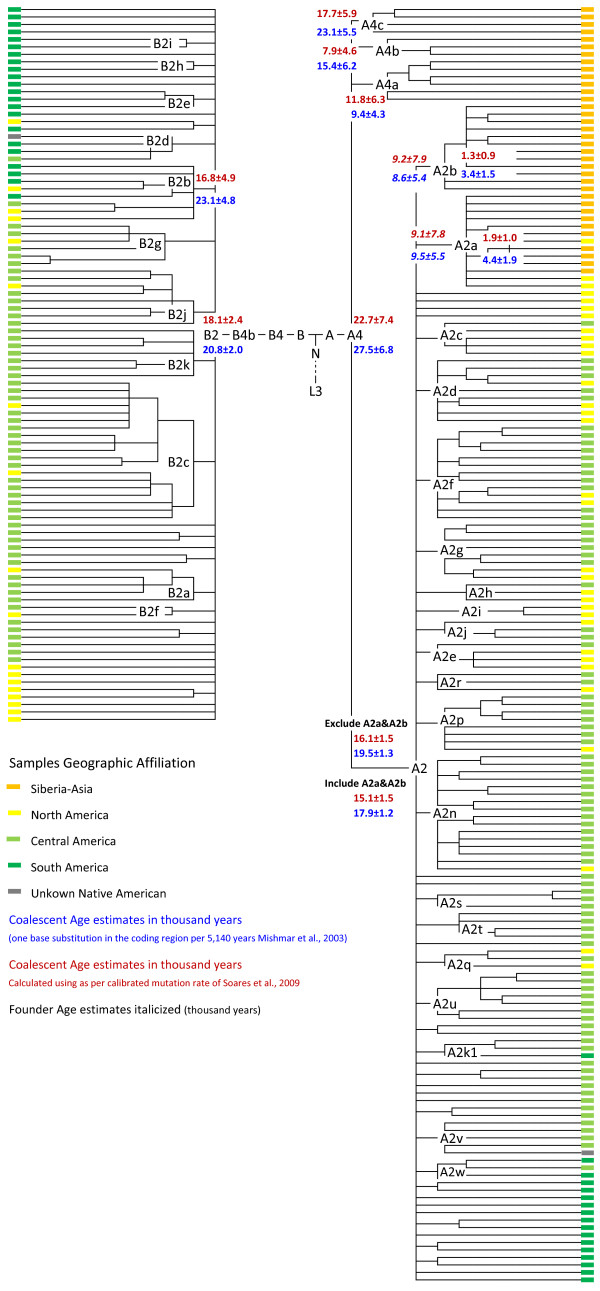
**Schematic representation of mtDNA phylogenetic tree of Native American haplogroups A2 and B2 and immediate Siberian-Asian sister clades (A2a, A2b, A4a, A4b and A4c)**. Coalescent age calculated in thousand years (ky) as per the slow mutation rate of Mishmar et al. [58] and as per calibrated mutation rate of Soares et al. [59] are indicated in blue and red color respectively. The founder age wherever calculated are italicized. The geographical locations of the samples are identified with colors. For more details see complete phylogenetic reconstruction in additional file 2 (panels A-B) and additional file 3.

**Figure 2:**
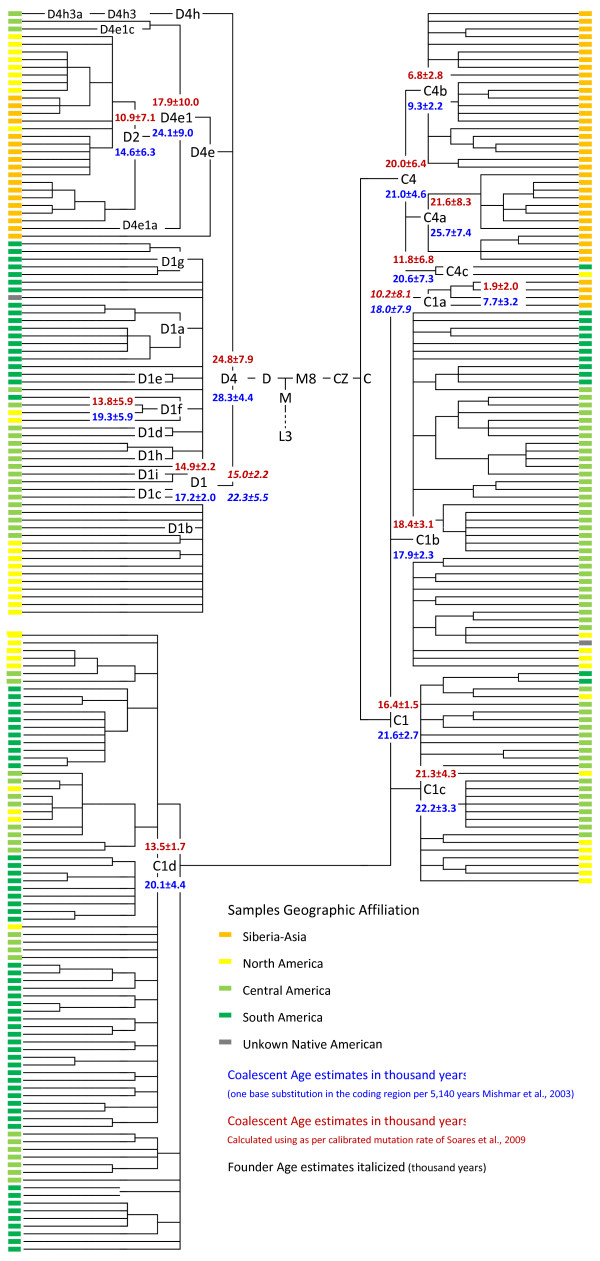
**Schematic representation of mtDNA phylogenetic tree of Native American haplogroups C1, C4c, D1, and D4e1c and their immediate Siberian-Asian sister clades (C1a, C4a, C4b, D2 and D4e1a)**. Coalescent age calculated in thousand years (ky) as per the slow mutation rate of Mishmar et al. [58] and as per calibrated mutation rate of Soares et al. [59] are indicated in blue and red color respectively. The founder age wherever calculated are italicized. The geographical locations of the samples are identified with colors. For more details see complete phylogenetic reconstruction in additional file 2 (panels C-D) and additional file 3.

The phylogenetic reconstruction and coalescent age estimates of the aforesaid 568 mitochondrial genomes is presented in additional file 2 (panels A-D) and summarized in Figure 1, Figure 2 and table 1.

**Table 1:** Haplogroup coalescence time estimates

				All substitutions			Synonymous transitions			Synonymous substitutions			
				
Haplogroup			*N*	*ρ*	*σ*	*T (ky)^a^*	*ρ*	*σ*	*T (ky)^b^*	*ρ*	*σ*	*T (ky)^c^*	*95% CI (ky)^d^*
**A4**			171	5.36	1.33	27.5 ± 6.8	2.86	0.94	19.4 ± 6.4	2.88	0.94	22.7 ± 7.4	8.2-37.2
	A2		158	3.48	0.24	17.9 ± 1.2	1.89	0.19	12.8 ± 1.3	1.92	0.19	15.1 ± 1.5	12.2-18.0
	A2*		133	3.80	0.25	19.5 ± 1.3	2.03	0.19	13.7 ± 1.3	2.05	0.19	16.1 ± 1.5	13.2-19.1
	A4a		6	1.83	0.83	9.4 ± 4.3	1.50	0.80	10.1 ± 5.4	1.50	0.80	11.8 ± 6.3	0-24.2
	A4b		3	3.00	1.20	15.4 ± 6.2	0.66	0.47	4.5 ± 3.2	1.00	0.58	7.9 ± 4.6	0-16.8
	A4c		4	4.50	1.06	23.1 ± 5.5	2.25	0.75	15.2 ± 5.1	2.25	0.75	17.7 ± 5.9	6.1-29.2
	**B2**		96	4.05	0.39	20.8 ± 2.0	2.21	0.31	14.9 ± 2.1	2.30	0.31	18.1 ± 2.4	13.3-22.9
		B2b	8	4.50	0.94	23.1 ± 4.8	2.13	0.62	14.4 ± 4.2	2.13	0.62	16.8 ± 4.9	7.2-26.3
**C4**			35	4.09	0.90	21.0 ± 4.6	2.54	0.81	17.2 ± 5.5	2.54	0.81	20.0 ± 6.4	7.5-32.5
	C4a		12	5.00	1.44	25.7 ± 7.4	2.75	1.05	18.6 ± 7.1	2.75	1.05	21.6 ± 8.3	5.4-37.8
	C4b		21	1.81	0.43	9.3 ± 2.2	0.86	0.36	5.8 ± 2.4	0.86	0.36	6.8 ± 2.8	1.2-12.3
	C4c**		2	4.00	1.41	20.6 ± 7.3	1.50	0.87	10.1 ± 5.9	1.50	0.87	11.8 ± 6.8	0-25.2
	**C1**		160	4.20	0.28	21.6 ± 2.7	2.056	0.19	13.9 ± 1.3	2.08	0.19	16.4 ± 1.5	13.5-19.3
		C1a	4	1.50	0.61	7.7 ± 3.2	0.25	0.25	1.7 ± 1.7	0.25	0.25	1.9 ± 1.9	0-5.8
		C1b	47	3.49	0.44	17.9 ± 2.3	2.34	0.39	15.8 ± 2.6	2.34	0.39	18.4 ± 3.1	12.4-24.4
		C1c	28	4.32	0.65	22.2 ± 3.3	2.64	0.54	17.9 ± 3.7	2.71	0.55	21.3 ± 4.3	12.8-29.8
		C1d	81	3.91	0.86	20.1 ± 4.4	1.70	0.21	11.5 ± 1.4	1.72	0.21	13.5 ± 1.7	10.2-16.8
**D4e1**			53	4.70	1.76	24.1 ± 9.0	2.28	1.27	15.4 ± 8.6	2.28	1.27	17.9 ± 10	0-37.5
	D2		50	2.84	1.23	14.6 ± 6.3	1.38	0.90	9.3 ± 6.1	1.38	0.90	10.9 ± 7.1	0-24.8
	D4e1c**		2	0.50	0.50	2.6 ± 2.6	*-*	-	-	-	-	-	-
	**D1**		49	3.35	0.39	17.2 ± 2.0	1.84	0.28	12.4 ± 1.9	1.90	0.28	14.9 ± 2.2	10.6-19.3
		D1f	4	3.75	1.15	19.3 ± 5.9	1.75	0.75	11.8 ± 5.1	1.75	0.75	13.8 ± 5.9	2.2-25.3

^a ^mutation rate: one base substitution in the coding region per 5, 140 years [58].

^b ^mutation rate: one synonymous transition per 6, 764 year [76].

^c ^Calculated using as per calibrated mutation rate of Soares et al. [59].

^d ^95% confidence interval: negative values are zeroed.

* Excluding branches A2a and A2b.

** Only two sequences hence coalescent age not reliable.

#### Haplogroup 'A2' and its sister clades

A total of 134 American sequences (49 published earlier and 85 from this study) belonging to pan-American haplogroup A2, form 42 distinct haplotypes, one nested within the Beringian subtype A2a and the other 41 largely autochthonous to geographical North, Central and South America, except Central American which shares a few branches with both North and South Americans. 12 of these 41 haplotypes were previously labeled A2c through A2q [57]. However, based on our phylogenetic reconstruction of the haplotypes, we changed the previously reported definitions of A2e, A2f and A2n. Clade A2e is now defined by only one substitution at (13708), A2f sequences share a back mutation at np 153, and A2n has a back mutation at 16111 (but not at 153), although it should be noted that the control region mutations are recurrent. The other new haplotypes with three or more sequences have been labeled A2r through A2w.

Of the two Beringian specific branches (A2a and A2b), A2a is the only sublineage shared by the Chukchi, Siberian and North American Eskimos, Aleuts, and Na-Dene populations. The A2a and A2b sublineages might have diverged from the rest of the American subtypes sometime between 17 to 9 kya, as suggested by the founder coalescent ages of A2a and A2b and the coalescent age of haplogroup A2 calculated using the mutation rates of Mishmar et al. [58] and Soares et al. [59]. The coalescent age of haplogroup A2 calculated using these two mutation rates over America specific lineages (19.5 ± 1.3 kya/16.1 ± 1.5 kya) indicates its early expansion into America. The haplogroup A2 shares the root A4 with the Siberia-Asia specific sister clades A4a, A4b and A4c. The coalescent age estimate of A4, as per mutation rates of Mishmar et al. [58] and Soares et al. [59], are 27.5 ± 6.8 kya and 22.7 ± 7.4 kya respectively.

#### Haplogroup 'B2'

The phylogeny of haplogroup B2 reveals a total of 36 haplotypes, with 9 previously defined subhaplogroups named B2a through B2i [57], and two new lineages, B2j and B2k, defined here by control region substitutions at 16278 and 152, respectively. The remaining haplotypes were not classified because they consist of only two or fewer representative sequences. Similar to the other pan American haplogroups, sublineages within the B2 haplogroup are specific to geographical regions (North, Central and South America). Only a few branches harbor sequences across geographical regions. The sub branch B2b by virtue of its early differentiation (coalescent age 23.1 ± 4.8 kya/16.8 ± 4.9 kya calculated as per [58] and [59] respectively) encompasses the North, Central and South America. The coalescent age estimates of B2 calculated as per [58] and [59] mutation rates are 20.8 ± 2.0 kya and 18.1 ± 2.4 kya respectively. The haplogroup B2 lacks any known immediate sister/ancestral clades in Siberia other then the B4b1 in South East Asia.

#### Haplogroup 'C1' and 'C4'

The C root is represented by two lineages in America: the pan American haplogroup C1; and the recently identified subhaplogroup 'C4c', found in only two sequences, an Ijka sample from South America [23] and a Shuswap speaker from North America [60]. The C4c subhaplogroup shares root C4 with two wide spread lineages in Siberia-Asia, subhaplogroup C4a (coalescent age 25.7 ± 7.4 kya/21.6 ± 8.3 kya calculated as per [58] and [59] respectively) and C4b (coalescent age 9.3 ± 2.2 kya/6.8 ± 2.8 kya calculated as per [58] and [59] respectively). The coalescence age estimates of the C4c (20.6 ± 7.3 kya/11.8 ± 6.8 kya calculated as per [58] and [59] respectively) are based on only two known sequences and are therefore not very reliable; coalescent age of root C4 (21.0 ± 4.6 kya/20.0 ± 6.4 calculated as per [58] and [59] respectively) suggest its early differentiation.

In the case of the more abundant and widely distributed haplogroup C1, three basal branches are ubiquitous in the Americas (C1b, C1c and C1d), and spread across the three major geographic regions. The fourth branch, C1a, is found in Siberian and East Asian populations. The C1c branch exhibits an older coalescent age of 22.2 ± 3.3 kya/21.3 ± 4.3 kya (calculated as per [58] and [59] respectively), harboring sublineages (e.g., C1c3) that are spread across the three major geographical regions. The coalescent age of C1b is estimated to be 17.9 ± 2.3kya/18.4 ± 3.1kya (calculated as per [58] and [59] respectively) and for C1d, it is 20.1 ± 4.4 kya/13.5 ± 1.7 kya (calculated as per [58] and [59] respectively). The estimated coalescent age of Siberian branch C1a is 7.7 ± 3.2 kya/1.9 ± 1.9 kya (calculated as per [58] and [59] respectively) which is considerably younger. However its founder age is estimated to be 18.0 ± 7.9 kya/10.2 ± 8.1 kya which is comparable to the American branches (after allowing for standard error). The estimated coalescent ages of the roots for C4 (21.0 ± 4.6 kya/20.0 ± 6.4) and C1 (21.4 ± 2.7 kya/16.4 ± 1.5) calculated as per mutation rates of Mishmar et al. [58] and Soares et al. [59], along with their subclades C4a and C1c, respectively, indicate that these Native American clusters diverged from Siberian-Asian sister clades sometime during the LGM peak.

#### Haplogroup 'D1', 'D4e'and 'D4h'

The phylogenetic reconstruction of 19 new and 60 published mitochondrial genomes from America and Siberia-Asia reveals that haplogroup 'D' is represented by at least three branches in America. The D1 haplogroup is found in high frequency throughout the Americas. D4h3a is rare but also widely distributed. We identified two Mexican American sequences belonging to a new sub branch named D4e1c, defined by the substitutions 14207 and 16092. This new branch has been defined in our tree based on the only two sequences, but an exception was made due to its phylogenetic importance. The D4e1c shares the root D4e1 (substitutions 3316 and 9536) with D2, which is a diverse lineage found from southern Siberia to Beringia [9,37,38,61-63] and D4e1a found in Japan [64]. Since D4e1c has only two known sequences, coalescent age estimates are not reliable. However coalescent age estimates of D4e1 24.1 ± 9.0 kya/17.9 ± 10.0 kya (calculated as per [58] and [59] respectively) suggests early divergence of D4e1c from its sister clades Siberian/Beringian D2 and Japanese D4e1a. The phylogeny of D1 reveals 25 haplotypes, all more or less autochthonous to respective geographical regions except sub branch D1f which harbors genomes from North, Central and South America. Based on its phylogeography and coalescent age (19.3 ± 5.9 kya/13.8 ± 5.9kya), D1f appears to have differentiated early. The overall coalescent age of D1 haplogroup is 17.2 ± 2.0 kya/14.9 ± 2.2 kya (calculated as per [58] and [59] respectively).

We did not find sequences belonging to rare haplogroup X2a in our sample, whereas the other rare haplogroup D4h3 is represented by only one sequence. A recent study [28], based on 22 X2a and 46 D4h3 complete mtDNA sequences concluded that although the coding region divergence of these haplogroups shows considerable overlap, their phylogeography in America is strikingly different. The haplogroup D4h3 is found along the Pacific coast; whereas X2a is restricted to northern North America and hence envisaged two different paths of arrival. The haplogroup D4h3 followed the Pacific coastal path just like other pan American haplogroups, whereas X2a might have arrived from Beringia through a path represented by the ice free corridor between the Laurentide and Cordilleran ice sheets [28]. We concentrated our further analysis on A2, B2, C1, C4c, D4e1c and D1 American haplogroups and their Siberian/Asian sister clades.

### Diversity indices and neutrality tests

Since we were interested in detecting the genetic consequences of demographic events that occurred during and after the peopling of the double continent, we computed nucleotide diversity and different estimators of the parameter θ = 2 N_e_μ to compare the amount of within population variability in Beringia, North America, Central America and South America. Also, in order to investigate the possibility of later expansions/migrations of Native American populations as suggested previously [28,65], we also performed the analysis by categorizing the Native American samples with respect to the four pan American haplogroups in the respective geographic regions.

The similar estimates of nucleotide diversity and *θ_π _*in North America (0.001747, 27.0), Central America (0.001735, 26.8) and South America (0.001834, 28.3) and the significantly negative value of Tajima's *D *and Fu's *Fs *(Table 2) suggest almost simultaneous (over a period of approximately 2.0 ky from South to North America) colonization of the three geographical regions, with rapid population expansion differentiating into more or less regional branches across the haplogroups (Figure 1 and 2). However the comparatively higher value of *θ_k _*(1338.1 and 917.5) and significantly negative Fu's *Fs *(-24.0, *p *= 0.000 and -23.8, *p *= 0.004) in North and Central America, respectively, may also suggest an excess of rare alleles/haplotypes. The value of *θ_k _*(413.5) in South America is comparatively low when compared to North and Central America.

**Table 2:** Diversity Indices and Neutrality tests for geographical region Beringia, North America, Central America and South America based on mtDNA coding region (np 577 - 16023) sequences.

Population/Geographic Region	*n^b^*	Nucleotide Diversity (SD)%^c^	*θ_k _*(95% CI)	*θ_s _*(SD)	*θ_π _*(SD)	Tejima's *D*	*P*- value *^d^*	Fu's *Fs*	*P*- value *^d^*
**Diversity in geographic regions**									
Beringia	66 *^e^*	0.0959 (0.0482)	14.198 (8.4 - 23.4)	11.136 (3.241)	14.811 (7.441)	1.021	0.892	0.448	0.645
North America *^f^*	73	0.1747 (0.0858)	1338.057 (469.4 - 4380.0)	56.465 (14.649)	27.006 (13.258)	-2.042	0.004	-24.041	0.000
Central America *^f^*	139*^a^*	0.1735 (0.0846)	917.489 (532.7 - 1664.5)	70.373 (16.098)	26.820 (13.079)	-2.195	0.000	-23.787	0.004
South America *^f^*	75	0.1834 (0.0899)	413.514 (203.6 - 908.3)	52.168 (13.563)	28.326 (13.890)	-1.847	0.011	-24.048	0.001
**Within haplogroup diversity in respective geographic regions**									
*HaplogroupA2*									
North America	29	0.0615 (0.0323)	415.445 (108.5 - 1745.1)	24.485 (7.837)	9.494 (4.986)	-2.569	0.000	-21.684	0.000
Central America	86	0.0495 (0.0258)	465.938 (239.7 - 972.2)	40.591 (10.376)	7.651 (3.991)	-2.816	0.000	-24.854	0.000
South America	17	0.0602 (0.0326)	141.445 (36.0 - 601.3)	21.001 (7.691)	9.294 (5.029)	-2.376	0.001	-9.819	0.001
*Haplogroup B2*									
North America	18	0.0668 (0.0358)	158.779 (40.6 - 673.9)	20.351 (7.364)	10.320 (5.525)	-2.471	0.000	-10.079	0.001
Central America	55	0.0539 (0.0281)	261.236 (120.8 - 614.9)	28.850 (8.115)	8.330 (4.346)	-2.598	0.000	-24.826	0.000
B2 South America	22	0.0586 (0.0313)	216.779 (55.9 - 916.5)	18.928 (6.565)	9.048 (4.832)	-2.415	0.000	-12.381	0.000
*Haplogroup C1*									
North America	14	0.0487 (0.0271)	82.112 (20.6 - 352.2)	13.836 (5.432)	7.516 (4.193)	-1.994	0.009	-5.519	0.005
Central America	61	0.0501 (0.0262)	221.898 (112.1 - 468.9)	27.351 (7.564)	7.741 (4.054)	-2.510	0.000	-24.908	0.000
South America	17	0.0527 (0.0288)	57.228 (18.5 - 196.2)	14.790 (5.520)	8.147 (4.451)	-1.908	0.017	-5.787	0.012
*Haplogroup D1*									
North America	12	0.0363 (0.0211)	69.779 (17.4 - 300.2)	10.596 (4.402)	5.606 (3.259)	-2.125	0.001	-7.565	0.000
Central America	17	0.0425 (0.0237)	141.445 (36.0 - 601.3)	12.719 (4.796)	6.574 (3.657)	-2.235	0.002	-12.457	0.000
South America	19	0.0491 (0.0268)	45.013 (16.5 - 133.6)	12.017 (4.436)	7.591 (4.141)	-1.488	0.047	-6.217	0.004

*^a ^*Randomly selected from total 219 sequences to overcome computational limitation.

*^b ^*Sample size.

*^c ^*Nucleotide diversity (standard deviation) multiplied with 100.

*^d ^*Significant *P-*values < 0.05 (for Tajima's *D*) and < 0.02 (for Fu's *Fs*).

*^e ^*Samples belonging to sub haplogroups (A2a, A2b and D2) in Chukchi, Eskimos, and Aleuts populations from Siberia and Commander Island.

*^f ^*Samples belonging to pan American haplogroups (A2, B2, C1 and D1) only

SD: Standard deviation

The low values of diversity indices (nucleotide diversity = 0.000959; *θ_π _*= 14.8) and non-significantly negative values of both neutrality tests (Tejima's *D *= 1.02; Fu's *Fs *= 0.448) in Beringia suggest very different demographic patterns to that of Native Americans. The analysis of within haplogroups diversity in the respective geographic region (Table 2), though not significantly different when considered with standard deviation, shows two contrasting patterns of distribution i.e. nucleotide diversity and *θ_π _*levels for A2 and B2 haplogroups that are highest in North America, followed by South America and the lowest observed for Central America. In contrast, haplogroups C1 and D1 show a cline from South America to North America. As evident in the few shared branches across geographical region (Figure 1 and 2), the recent gene flow from the North and South might have resulted in the low diversity indices and higher values of *θ_k _*in the Central American populations. The high values of diversity indices (nucleotide diversity = 0.000615 and *θ_π _*= 9.5) along with high *θ_k _*(415.4) and highly significant negative Fu's *Fs *(-21.7, *p *= 0.00) of haplogroup A2 in North America may indicate a secondary expansion of the A2 haplogroup following the path represented by the ice free corridor between the Laurentide and Cordilleran ice sheets. A similar path for the expansion of haplogroup X2a in America has been suggested previously [28].

### Bayesian skyline plot analysis

**Figure 3:**
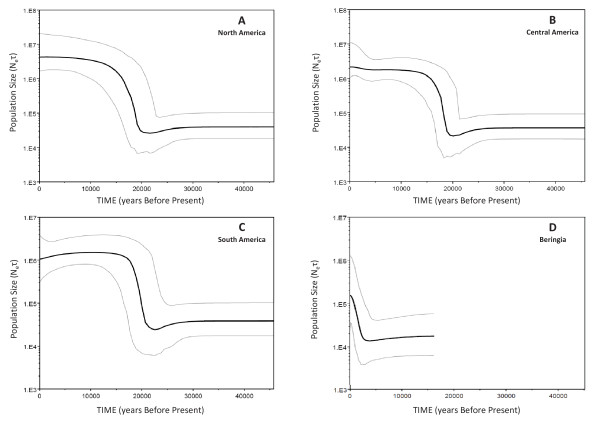
**Bayesian skyline plot showing Effective population size (*N_e_*τ) through time derived from mtDNA coding region (np 577 - 16023) sequences**. The thick solid line is the median estimate and the thin lines show the 95% highest posterior density limits. In this analysis *N_e_τ*, where *Ne *is the population of breeding females and *τ *is the generation length is plotted on Y axis. Time estimates obtained using a log-normal relaxed clock with the standard substitution rate of 1.26 × 10^-8 ^sites per year [58] are plotted on X axis and limited to < 45 kya. Panel 'A' - The Bayesian skyline plot (*m *= 10) for North America (*n *= 75). Panel 'B' - The Bayesian skyline plot (*m *= 10) for Central America (*n *= 151, randomly selected from total 215 sequences). Panel 'C' - The Bayesian skyline plot (*m *= 10) for South America (*n *= 76). Panel 'D' - The Bayesian skyline plot (*m *= 10) for Beringia (*n *= 66).

The above results suggest a complex demographic history associated with the colonization of the three geographic regions and of the Americas as a whole. We used Bayesian skyline plot analysis [66] to visually illustrate the change in effective population size in Beringia and three geographical regions of America. In this analysis, *N_e_τ *(where *N_e _*is the population of breeding females and *τ *is the generation length) is unaffected by the different proposals for the actual number of founder lineages among present day populations [66]. The Bayesian skyline plots presented in Figure 3 (panels A-D) show that Beringian populations (Chukchi and Eskimos including the Aleutian Islands) suffered a gradual population decline following their divergence from Native Americans and Siberian ancestors (~15-10 kya) and are establishing moderate growth ~2.5 kya. In contrast, a pronounced population growth starting ~21 kya (95% CI: 23-19 kya) in South America and ~19 kya (95% CI: 21-17 kya) in North and Central America, continuing up ~16-15 kya in South and Central America and a little prolonged ~14-12 kya in North America has been observed. As reported earlier [24], each region in our analysis identifies a moderate population bottle neck just before the manifold rapid growth, which probably suggests a time frame of Native American founder's divergence and differentiation from their Asian ancestors sometime around LGM and then rapid expansion into America.

## Discussion

**Figure 4:**
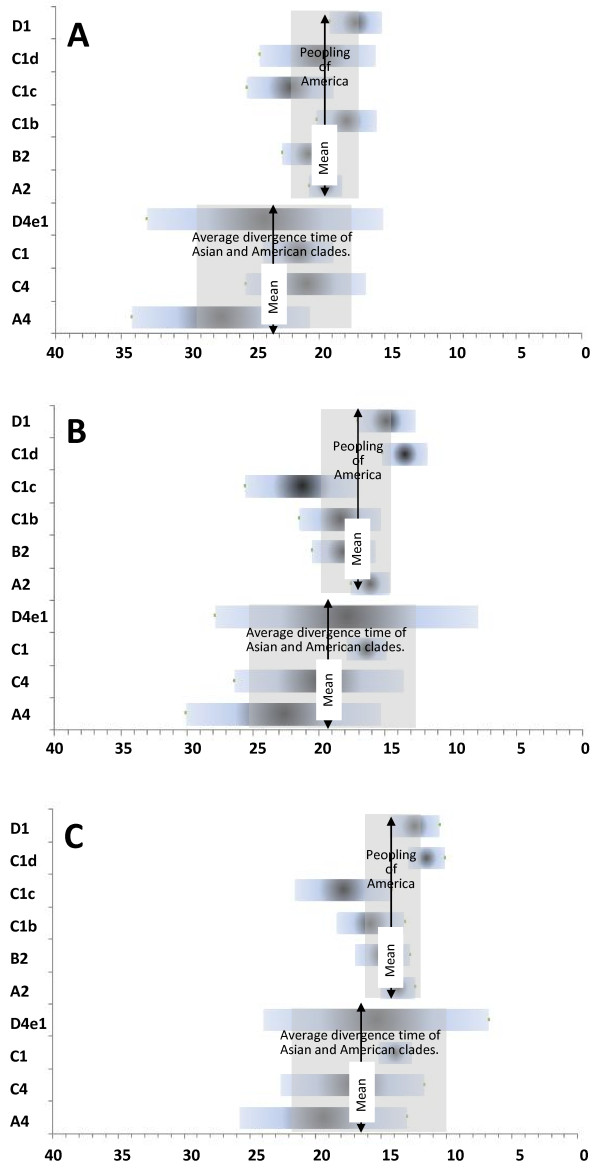
**Schematic presentation of the average divergence of the American clades from Siberian-Asian sister clades and their dispersal into the America**. Horizontal bars indicate the coalescent age estimates (at the center) ± Standard errors for each haplogroup shown on the Y axis. The average divergence time and the average time of the peopling of America across the haplogroups are also superimposed. X axis shows the time in thousand years ago. The coalescent estimates were calculated by Rho (ρ) statistics and three different mutation rates (i) panel 'A': one base substitution (i.e. one mutation other than indel) in the coding region (577 - 16023) per 5, 140 years [58], (ii) panel 'B': calibrated mutation rate of Soares et al. [59] based on all synonymous substitutions, (iii) panel 'C': one synonymous transition per 6, 764 year [76]

Our analysis of a large data set of 568 mitochondrial genomes (215 this study and 353 from published sources; see additional file 1 for details) from North, Central and South America, as well as Beringia and Siberia-Asia shows that the coalescent age estimates of shared roots calculated as per the mutation rates of Mishmar et al. [58] and Soares et al. [59] between Siberian-Asian and Native American lineages i.e. A4 (27.5 ± 6.8 kya/22.7 ± 7.4 kya), C1 (21.4 ± 2.7 kya/16.4 ± 1.5 kya), C4 (21.0 ± 4.6 kya/20.0 ± 6.4 kya), and D4e1 (24.1 ± 9.0 kya/17.9 ± 10.0 kya) average around LGM when considered together with their standard deviations (Figure 4 panel A and B). These coalescent estimates along with the phylogeography of pan American haplogroups and Siberian-Asian sister clades strongly suggest that American founders diverged from their Siberian-Asian progenitors sometime during LGM. It is most likely that the hostile conditions during the LGM (centered at ~21.0 kya and extending from at least 23.0 to 19.0 kya) [67], caused by extensive glaciations, with a large ice lobe extending eastwards across northern Siberia [68,69] might have forced some populations back into southern Siberian LGM refuge, plausibly in Altai-Sayan and/or mid-lower Amur region [35,38] and the American founders to the east into Beringia. A swift expansion further south into the American continent occurred right after the LGM [24,65], most plausibly around 20-16 kya (Figure 4 panel A and B), as suggested by the coalescent age estimates of pan American haplogroups (A2:19.5 ± 1.3 kya/16.1 ± 1.5 kya, B2:20.8 ± 2.0 kya/18.1 ± 2.4 kya, C1:21.4 ± 2.7 kya/16.4 ± 1.5 kya and D1:17.2 ± 2.0 kya/14.9 ± 2.2 kya) calculated as per the mutation rates of Mishmar et al. [58] and Soares et al. [59] and estimates of populations expansions using Bayesian Skyline approach (Figure 3 panels A-C). The estimated average time difference between the divergence of Native Americans from their Siberian-Asian precursors and the expansion of founding population into America is less than 5.0 ky irrespective of the mutation rate used (Figure 4 panel A-C). The hostile conditions during LGM are also evident in the compilation of the records of fossil woody material or other plant macrofossils across northern Siberia i.e. records become progressively rarer right after 25 kya and then are absent completely for several thousand years in the period that includes the LGM [70,71]. Given that the opening of the ice free corridor is dated not earlier than ~14 kya, a coastal (Pacific) route would have been the only option for initial expansion [24,31-33]. Our data also show that the expansion of pan American haplogroups within the double continent is far more complex, contrary to the uniformity assumed by the BIM models and its variants [23-26]. The earliest expansion observed in the population reaching South America support the pre-Clovis occupation of the New World via the coastal route right after LGM. The expansion in North America occurred after the LGM (~19 kya) indicating a still prevailing LGM condition in the north. These expansions, separated by time and space and perhaps involving pan American lineages differentially, are responsible for a complex pattern of mtDNA diversity within the haplogroups A2, B2, C1, and D1 in the three geographical regions of America. Some intra-haplogroup variation, at least differentiation of sub branches B2b, C1b, C1c, C1d and D1f, already existed in American founders before expansion into the Americas. The population bottleneck, also reported previously [24], though moderate during this period (LGM) and a many fold rapid expansion right after LGM (~19 kya) perhaps better explains the few founder types in America.

The phylogeography and diversity within haplogroup C1 shows a cline south to north in America. However C1 is subdivided in the three pan American braches (C1b, C1c, C1d) [23,24,65,72] and a Siberian-Asian representative C1a [38,62,64,73]. The subhaplogroup C1a, which shares root substitutions with American sub branches (C1b, C1c and C1d), is found in Japan [64] and southern Siberia [38,62,73]. Finding C1a in America or Beringia could help to explain the presence of C1a in Siberia-Asia as a result of a back migration from Beringia or America, a scenario enforced in BIM by Tamm et al. [23] to explain origin of haplogroup C1 in Beringia and its phylogeography. Yet, to date this has not been achieved even after large sampling from this area [23,24,28,37-39,58,60-62,65,72-76]. Therefore, one still cannot rule out the most parsimonious scenario that haplogroup C1 originated in Siberia-Asia [38,62]. It is also worth noting that even though C1a is found across diverse populations in Siberia-Asia, it is rare; whereas C1, which differentiated into C1b, C1c and C1d, is found in high frequency in America, and its phylogeography and coalescent age in America is very similar to the other pan American haplogroups (A2, B2 and D1) [23,24,65]. Therefore disappearing C1a from the expanding population in America appears unlikely given that it would require disappearance of a lineage from an expanding population in an unpopulated territory, a demographic situation in which loss of lineage due to genetic drift is minimized [77]. Furthermore back migration of only C1a from Beringia to Siberia-Asia as suggested in BIM by Tamm et al. [23], when all the pan American haplogroup existed in a stable, plausibly expanding population in Beringia seems less parsimonious.

In addition to C1, another founder lineage 'C4c' has been defined recently in Native Americans [23,60], which shares root 'C4' with the diverse Siberian-Asian sister clades C4a and C4b. The coalescent age estimates of C1 (21.4 ± 2.7 kya/16.4 ± 1.5 kya) and C4 (20.7 ± 4.3 kya/20.0 ± 6.4 kya) calculated as per [58] and [59] further support a recent divergence of American founders from their Siberian-Asian progenitors.

The haplogroup A2 coalescent age (17.9 ± 1.2 kya/16.1 ± 1.5 kya calculated as per [58] and [59] respectively), phylogeography and diversity distribution in America and Beringia, has been used to suggest its in-situ origin in American founders plausibly in the Beringian-Alaskan region [38]. However this diversity is restricted to only two branches (A2a and A2b) in Beringia. Therefore differentiation of its precursor in Siberia-Asia or soon after the divergence of Native American founders from their Siberian-Asian precursor cannot be ruled out. The time window for such differentiation would have been any time between the age of its shared root A4 (27.5 ± 6.8 kya/22.7 ± 7.4 kya calculated as per [58] and [59] respectively) with Siberian-Asian sister clades (A4a, A4b, A4c) and its own American coalescent age (19.5 ± 1.3 kya/16.4 ± 1.5 kya calculated as per [58] and [59] respectively), or more plausibly ~21-19 kya marked by the first expansion of modern human into America [24,65]. The diversity distribution along with demographic parameter also indicates that there could be more than one expansion of A2 in North America plausibly following two different paths, one Pacific coastal path just like the other pan American haplogroups and another path represented by the ice free corridor between the Laurentide and Cordilleran ice sheets, similar to X2a [28,65]. The environmental and paleoecological data, indicates that such a path existed and was represented by the ice-free corridor between the Laurentide and Cordilleran ice sheets, which opened approximately 15 kya [78] or possibly was never completely closed [79]. Through such a corridor, small glacial-refuge areas have been recently identified [80].

The phylogeography and within haplogroup diversity of haplogroup B2 is very similar to haplogroup A2 and supports a major expansion from North America to South America. So far haplogroup B2 lacks any known immediate sister/ancestral clades in Siberia other then the B4b1 in South East Asia, and it remains unclear whether the B2, dating to 20.8 ± 2.0 kya/18.1 ± 2.4 kya, has evolved *in situ *in American founders after their divergence from Siberian-Asian ancestors or Siberia-Asia itself.

The D4 clusters coalescent age (28.3 ± 4.4 kya/24.8 ± 7.9 kya calculated as per [58] and [59] respectively), represents the divergence age of its sub branches, D1 found only in America and D4e1 and D4h3 having distributed from Asia to America, suggest a more complex process of differentiation. The within haplogroup diversity of haplogroup D1 showing a cline south to north in America and having at least one sub branch 'D1f', already differentiated before the expansion and numerous later regional sub branches strongly suggest its early differentiation and a sizable population with basal D1 prior to expansion into America. Contrary to the absence of any known progenitor in Siberia-Asia, a Siberian-Asian origin of D1 cannot be ruled out [35,62]. The D4e1 is represented by Japanese branch D4e1a in Asia [64], D2 in Siberia/Beringia and by our newly discovered D4e1c branch in America. D2 constitutes two different clusters with contrasting geographic distribution. The cluster D2a is found in high frequency among Chukchi, Aleuts, and Eskimos [36-39,61-63], whereas the second cluster D2b is reported in Buryat, Khamnigan and Yakut populations [38]. The phylogeography of D2 and the coalescent age estimate (14.6 ± 6.3 kya/10.9 ± 7.1 kya calculated as per [58] and [59] respectively) may suggest its Southern Siberian origin and later expansion to Beringia. But American clade D4e1c, though rare, known by only two sequences from our current work, diverge from the root D4e1 and the coalescent age of D4e1 (24.1 ± 9.0 kya/17.9 ± 10.0 kya calculated as per [58] and [59] respectively), strongly suggest that D4e1c was the part of initial colonization of Americas.

## Conclusions

Our analysis of a data set of 568 mitochondrial genomes from North, Central and South America, as well as Beringia and Siberia-Asia suggest that American founders diverged from their Siberian-Asian progenitors sometime during LGM and expanded into America soon after the LGM peak (~20-16 kya). Further, time between the Native American divergence from Siberian-Asians to the expansion into America was shorter than the previous estimates. The phylogeography of haplogroup C1 suggest that this American founder haplogroup differentiated in Siberia-Asia. Although it is not clear for the haplogroup B2, haplogroups A2 and D1 might have differentiated soon after the Native American founder's divergence. A moderate population bottle neck in American founder populations just before the expansion (Figure 3) most plausibly resulted in few founder types in America. The similar estimates of the diversity indices and Bayesian skyline analysis in North America, Central America and South America suggest almost simultaneous (over a period of approximately 2.0 ky from South to North America) colonization of these geographical regions with rapid population expansion differentiating into more or less regional branches across the pan American haplogroups A2, B2, D1 and the C1b, C1c, C1d subhaplogroups of C1. However, some sub branches (B2b, C1b, C1c, C1d and D1f) already existed in American founder haplogroups before expansion into the Americas.

## Methods

There have been considerable advances in the understanding of Native American mtDNA phylogeography and the peopling processes of the American continent in recent years; however this has been based largely on mitochondrial genomes sampled from North and South American populations, with very few coming from southern North America and Central America [23,24,58,65,74-76]. Therefore, in order to address this insufficiency, we have completely sequenced mtDNA of 384 Mexican American maternal lines from our San Antonio Family Heart Study (SAFHS). Most Mexican Americans are the descendants of the Indigenous peoples of Mexico and/or Europeans [40], especially Spaniards and Mexico is considered as the southern tip of North America in general. Considering the pre-modern societies such as the Maya, who thrived in one cultural sphere that spread from El Salvador, Cozumel and Central Mexico, and into Veracruz, Honduras and Guatemala [81]; we consider our population sample has strong ethnic ties to Central America and therefore for the analysis combined with very few Central American mitochondrial genomes published previously.

### Population samples

Mexican Americans account for more than 12.5% of the United States' population: 30.7 million Americans listed their ancestry as Mexican as of 2008, forming about 64% of all Hispanics and Latinos in the United States [82,83].

We have sampled 1, 236 Mexican American individuals organized into 42 extended families from the SAFHS [84]. From these extended pedigrees we selected 384 pedigree founders, representing all maternal lines in the family sample, for complete mtDNA sequencing. All participants gave informed consent and all protocols were approved by the Institutional Review Board of the University of Texas Health Science Center at San Antonio (San Antonio, TX).

As reported previously [53,54] and also observed in our study, mtDNA sequence showed some of the Mexican American individuals were of European and African ancestry due to recent admixture in historic times. Additionally, some of the founder individuals sequenced were related (from the same maternal line: based on our pedigree information). To focus our analyses, we removed all such genomes from this study and analyzed our 215 newly sequenced unrelated mitochondrial genomes belonging to Native American ancestry along with 353 from published source belonging to Native American haplogroups (A2, B2, C1 and D1) and their Asian sister clades (A4, C4 and D4e) [23,24,38,58,60-62,64,65,72-76,85]. The mtDNA sequences of Herrnstadt et al. [75] and Kivisild et al. [76] lack control region information. The 63 published source genomes [72], which were sequenced selectively for subhaplogroup C1d, were excluded from diversity and Bayesian skyline analysis because preselected sequences of single subhaplogroup would have skewed the results of these demographic parameters. See additional file 1 for more details on the mtDNA sequences used in this study.

### Molecular analysis

The DNA was extracted from collected white blood cell from 10 ml blood samples using standard phenol-chloroform methods [86] with minor modifications. For complete mtDNA resequencing we used the mitoSEQr™ kit (Applied Biosystems) designed to amplify human mitochondrial DNA in 46 fragments using universal polymerase chain reaction (PCR) conditions. However we had mixed success with some of the fragments, so the resequencing of such regions of the genome was completed using alternate PCR primers and conditions of Rieder et al. [87]. Successful PCR amplification was verified on 1% ethidium bromide stained agarose gels, and purified using ExoSap-IT to remove excess primers and deoxynucleotide triphosphates (GE Healthcare). The amplicons were then used as templates in cycle sequencing reactions using the BigDye Terminator v3.1 Cycle Sequencing Ready Reaction Kit (Applied Biosystems), according to the manufacturer's instructions. The cycle sequencing products were purified using BigDye XTerminator Purification Kit (Applied Biosystems) to remove unincorporated BigDye and primer and then analyzed on an Applied Biosystems 3730 DNA Analyzer. Both strands were sequenced to promote resolution of polymorphisms. Applied Biosystems supplied DNA Sequencing Analysis Software version 5.1.1 was used for first pass base calling quality assessment. Comprehensive contig assembly and sequence alignment was performed in SeqScape Software version 2.6 (Applied Biosystems). Mutations were scored relative to the revised Cambridge Reference Sequence (rCRS) [88] with each deviation confirmed by manual checking of electropherograms. All (n = 215) mtDNA complete genome sequences used in this study have been submitted to GenBank (accession numbers HQ012049-HQ012263).

### Statistical analysis

#### Phylogeny reconstruction and age estimation

Besides our newly sequenced 215 unrelated mitochondrial genomes of Native American ancestry, 353 additional published sequences belonging to Native American haplogroups (A2, B2, C1 and D1) and their Siberian-Asian sister clades (A4, C4 and D4e) as detailed in additional file 1 were employed for tree reconstruction. The phylogenetic trees of each of the pan American mtDNA haplogroups and their sister clades were reconstructed from median joining networks rooted to L3, using NETWORK 4.2.0.1 software [89]. The trees were checked manually to resolve homoplasies. Since we lack control region information for previously published sequences of Herrnstadt et al. [75] and Kivisild et al. [76], we used slow evolving coding region information for coalescent age estimates. The coalescent age estimates were calculated by Rho (ρ) statistics [90] and three different mutation rates: (i) one base substitution (i.e. one mutation other than indel) in the coding region (577 - 16023) per 5, 140 years [58]; (ii) one synonymous transition per 6, 764 year [76]; and (iii) calibrated mutation rate of [59] based on all synonymous substitutions. All of these mutation rates are calibrated on the basis of an assumed human-chimp split. Standard errors for coalescence estimates were calculated as per Saillard et al. [90]. The coalescent age estimates using the three aforesaid mutation rates are presented in table 1. It has been observed in our analysis and previously [23] that coalescent estimates based on the effectively faster rate of Kivisild et al. [76], yields younger age for most Native American haplogroups than the well documented archaeological date of modern human occupation at Monte Verde site in southern South America [30]. The mutation rates of Mishmar et al. [58] and Soares et al. [59] yielded higher and similar coalescent ages which fit well with the archaeological estimates of modern human occupation at Monte Verde site in southern South America [30]. Therefore, we largely based our inferences on the coalescent age estimates of these two mutation rates.

#### Diversity indices and neutrality tests

We computed nucleotide diversity and different estimators of the parameter *θ *= 2 N_e_μ to compare the amount of within population variability in Beringia, North America, Central America and South America: (1) *θ_k _*[91], based on the number of observed alleles (*k*), (2) *θ_S _*[92], based on the number of observed segregating sites (*S*), and (3) *θ_π _*[93], based on the mean number of pair-wise differences between sequences (π). We used Tajima's *D *[94] and Fu's *Fs *[95] statistics to investigate population expansion events. To investigate any event of second expansion/migration affecting or involving haplogroups differentially, we also performed the analysis by categorizing the Native American samples with respect to four pan American haplogroups in the respective geographic regions. The aforesaid diversity indices and neutrality test were performed on the slow evolving mtDNA coding region (position 577-16023) using the software package ARLEQUIN version 3.1 [96].

#### Bayesian skyline plot analysis

We used Bayesian skyline model of effective population size [66] to visually illustrate the demographic history of Beringian and Native American populations from the Most Recent Common Ancestor (MRCA). Effective population size is a compound population genetic parameter generally considered linearly proportional to census population size, the population of breeding females in this analysis. It is influenced by many factors, including local extinction, re-colonization and various forms of nonrandom mating [97]. The model assumes regional isolation and no phylogenetic structure priori, such as the existence of haplogroups or the number of founding haplotypes. Estimates of effective populations over time for geographic regions Beringia (*n *= 66), North America (*n *= 75), Central America (*n *= 151; randomly selected from 215 to overcome computation limitation in BEAST), and South America (*n *= 76), were derived from coding region mtDNA sequences. We considered Chukchi and Eskimos sampled from Siberia, and Aleuts sampled from Commander Island as the representative Beringian populations in this analysis. The analysis was carried out assuming HKY+G model and a log-normal relaxed clock [98] with the standard substitution rate of 1.26 × 10^8 ^sites per year [58] using Markov Chain Monte Carlo (MCMC) [99] sampling with 10 groups (m = 10). The software packages BEAST v1.4.8 [100] and Tracer v1.4.1 [101] used in this analysis are available from http://beast.bio.ed.ac.uk/. The plots were obtained using a stepwise (constant) model. The analysis was run for 30 million iterations for Beringia, North and South America and 40 million iterations for Central America with the first 10% discarded as burn-in in each case, genealogies and model parameters were sampled at every 1000 iterations thereafter.

## List of Abbreviations

LGM: Last Glacial Maximum; kya: Thousand Years Ago; mtDNA: Mitochondrial DNA; BIM: Beringian Incubation Model; SAFHS: San Antonio Family Heart Study; PCR: Polymerase Chain Reaction; rCRS: revised Cambridge Reference Sequence; MRCA: Most Recent Common Ancestor; MCMC: Markov Chain Monte Carlo.

## Authors' contributions

SK, CB and JEC performed the complete mtDNA sequencing and sequence alignments. SK performed the database search and phylogenetic analysis. SK and JEC drafted the manuscript. MZ and PEM provided input into the analysis and helped to improve the manuscript. JB and JEC conceived the study, participated in its design and coordination, and also helped to improve the manuscript. All authors read and approved the final manuscript.

## Supplementary Material

Additional file 1 **Details of mitochondrial genomes used in the present study**. The file contains a table showing ethnographic details, GenBank accession numbers and references of the mitochondrial genome sequences used in the present study. Click here for file

Additional file 2 **The detailed mtDNA phylogenetic tree of Native American haplogroups and their immediate Siberian-Asian sister clades (panel A-D)**. The file contains figures showing phylogenetic reconstruction of 568 complete mitochondrial DNA sequences (Maximum parsimony tree), belonging to Native American haplogroups and Siberian-Asian sister clades presented in four panels "A-D" (see additional file 3 for more details). Click here for file

Additional file 3 **Figure Legends for additional file **2. The file contains details on phylogenetic reconstruction, sequences and mtDNA mutation rates used for coalescent age estimates in additional file 2. Click here for file
