# Supplementary material for: Large scale mitochondrial sequencing in Mexican Americans suggests a reappraisal of Native American origins
Source: BMC Evol Biol. 2011 Oct 7;11:293. doi: 10.1186/1471-2148-11-293 (PMC3217880; doi:10.1186/1471-2148-11-293)
Supplement: Additional file 1 — Details of mitochondrial genomes used in the present study. The file contains a table showing ethnographic details, GenBank accession numbers and references of the mitochondrial genome sequences used in the present study. [file 1471-2148-11-293-S1.DOC]

Additional file 1. Details of mitochondrial genomes used in the present study.

| **S. No.** | **HG** | **GenBank** | **Original ID** | **Geographic or** | **Region** | **Reference** |
| --- | --- | --- | --- | --- | --- | --- |
|  |  | **Acc. No.** |  | **Ethnic Origin** |  |  |
| 1 | A4c | EF153819 | Khamnigan66 | Khamnigan | Siberia | Derenko et al., 2007 |
| 2 | A4c | EF153787 | Buryat406 | Buryat | Siberia | Derenko et al., 2007 |
| 3 | A4c | EF153795 | Buryat575 | Buryat | Siberia | Derenko et al., 2007 |
| 4 | A4c | EF153791 | Buryat523 | Buryat | Siberia | Derenko et al., 2007 |
| 5 | A4c | EF153775 | Altai-Kizhi178 | Altai-Kizhi | Siberia | Derenko et al., 2007 |
| 6 | A4b | EF153771 | Buryat442 | Buryat | Siberia | Derenko et al., 2007 |
| 7 | A4b | EF397560 | Evenk42 | Evenk | Siberia | Derenko et al., 2007 |
| 8 | A4b | AY519488 | Mansi1A1 | Mansi | Siberia | Starikovskaya et al., 2005 |
| 9 | A4a | EF153792 | Buryat552 | Buryat | Siberia | Derenko et al., 2007 |
| 10 | A4a | EF153774 | Altai-Kizhi163 | Altai-Kizhi | Siberia | Derenko et al., 2007 |
| 11 | A4a | EF153799 | Buryat627 | Buryat | Siberia | Derenko et al., 2007 |
| 12 | A4a | EF153783 | Buryat390 | Buryat | Siberia | Derenko et al., 2007 |
| 13 | A4a | EF153833 | VN65 | Russian | Siberia | Derenko et al., 2007 |
| 14 | A4a | AP008617 | ON125 | Japanese | Asia | Tanaka et al., 2004 |
| 15^#^ | A2b | EF153806 | Chukchi5 | Chukchi | Chukotka | Derenko et al., 2007 |
| 16^#^ | A2b | EU482341 | ChuK40 | Chukchi | Chukotka | Volodko et al., 2008 |
| 17^#^ | A2b | EU482345 | Chuk104 | Chukchi | Chukotka | Volodko et al., 2008 |
| 18^#^ | A2b | EU482346 | Chuk105 | Chukchi | Chukotka | Volodko et al., 2008 |
| 19^#^ | A2b | AF346971 | Chukchi | Chukchi | Chukotka | Ingman et al., , 2000 |
| 20^#^ | A2b | EU482350 | Esk3 | Eskimo | Chukotka | Volodko et al., 2008 |
| 21^#^ | A2b | EF153827 | Koryak39 | Koryak | Chukotka | Derenko et al., 2007 |
| 22^#^ | A2b | EF153807 | Chukchi6 | Chukchi | Chukotka | Derenko et al., 2007 |
| 23^#^ | A2b | EU482353 | Esk14 | Eskimo | Chukotka | Volodko et al., 2008 |
| 24^#^ | A2b | EU482358 | Esk45 | Eskimo | Chukotka | Volodko et al., 2008 |
| 25^#^ | A2b | EF153809 | Chukchi11 | Chukchi | Chukotka | Derenko et al., 2007 |
| 26^#^ | A2b | EU482347 | Chuk106 | Chukchi | Chukotka | Volodko et al., 2008 |
| 27^#^ | A2a | EF153808 | Chukchi9 | Chukchi | Chukotka | Derenko et al., 2007 |
| 28^#^ | A2a | EU482349 | Esk1 | Eskimo | Chukotka | Volodko et al., 2008 |
| 29^#^ | A2a | EU482342 | Chuk101 | Chukchi | Chukotka | Volodko et al., 2008 |
| 30^#^ | A2a | EU482338 | Chuk22 | Chukchi | Chukotka | Volodko et al., 2008 |
| 31^#^ | A2a | EU482344 | Chuk103 | Chukchi | Chukotka | Volodko et al., 2008 |
| 32^#^ | A2a | EU482355 | Esk19 | Eskimo | Chukotka | Volodko et al., 2008 |
| 33 | A2a | EU095540 | Eskimo29 | Eskimo | Siberia | Tamm et al., 2007 |
| 34^#^ | A2a | EU482343 | Chuk102 | Chukchi | Chukotka | Volodko et al., 2008 |
| 35^#^ | A2a | EU482351 | Esk5 | Eskimo | Chukotka | Volodko et al., 2008 |
| 36^#^ | A2a | EU482352 | Esk13 | Eskimo | Chukotka | Volodko et al., 2008 |
| 37^#^ | A2a | EF153803 | Chukchi1 | Chukchi | Chukotka | Derenko et al., 2007 |
| 38 | A2a | EU095547 | Selkup94 | Selkup | Siberia | Tamm et al., 2007 |
| 39 | A2a | EU095526 | Apache514 | Apache | North America | Tamm et al., 2007 |
| 40 | A2 | EU095538 | Dogrib39 | Dogrib | North America | Tamm et al., 2007 |
| 41 | A2 | EU431080 | IAB_D6 | ** | North America | Achilli et al., 2008 |
| 42 | A2 | HQ012049 | MA001 | Mexican American | Central America | Present Study |
| 43 | A2 | HQ012050 | MA005 | Mexican American | Central America | Present Study |
| 44 | A2 | HQ012051 | MA009 | Mexican American | Central America | Present Study |
| 45 | A2 | HQ012052 | MA010 | Mexican American | Central America | Present Study |
| 46 | A2 | HQ012053 | MA012 | Mexican American | Central America | Present Study |
| 47 | A2 | HQ012054 | MA014 | Mexican American | Central America | Present Study |
| 48 | A2 | HQ012055 | MA017 | Mexican American | Central America | Present Study |
| 49 | A2 | HQ012056 | MA019 | Mexican American | Central America | Present Study |
| 50 | A2 | HQ012057 | MA023 | Mexican American | Central America | Present Study |
| 51 | A2 | HQ012058 | MA025 | Mexican American | Central America | Present Study |
| 52 | A2 | HQ012059 | MA028 | Mexican American | Central America | Present Study |
| 53 | A2 | HQ012060 | MA033 | Mexican American | Central America | Present Study |
| 54 | A2 | HQ012061 | MA038 | Mexican American | Central America | Present Study |
| 55 | A2 | HQ012062 | MA039 | Mexican American | Central America | Present Study |
| 56 | A2 | HQ012063 | MA042 | Mexican American | Central America | Present Study |
| 57 | A2 | HQ012064 | MA043 | Mexican American | Central America | Present Study |
| 58 | A2 | HQ012065 | MA047 | Mexican American | Central America | Present Study |
| 59 | A2 | HQ012066 | MA048 | Mexican American | Central America | Present Study |
| 60 | A2 | HQ012067 | MA054 | Mexican American | Central America | Present Study |
| 61 | A2 | HQ012068 | MA060 | Mexican American | Central America | Present Study |
| 62 | A2 | HQ012069 | MA062 | Mexican American | Central America | Present Study |
| 63 | A2 | HQ012070 | MA063 | Mexican American | Central America | Present Study |
| 64 | A2 | HQ012071 | MA067 | Mexican American | Central America | Present Study |
| 65 | A2 | HQ012072 | MA070 | Mexican American | Central America | Present Study |
| 66 | A2 | HQ012073 | MA075 | Mexican American | Central America | Present Study |
| 67 | A2 | HQ012074 | MA077 | Mexican American | Central America | Present Study |
| 68 | A2 | HQ012075 | MA080 | Mexican American | Central America | Present Study |
| 69 | A2 | HQ012076 | MA083 | Mexican American | Central America | Present Study |
| 70 | A2 | HQ012077 | MA086 | Mexican American | Central America | Present Study |
| 71 | A2 | HQ012078 | MA091 | Mexican American | Central America | Present Study |
| 72 | A2 | HQ012079 | MA093 | Mexican American | Central America | Present Study |
| 73 | A2 | HQ012080 | MA095 | Mexican American | Central America | Present Study |
| 74 | A2 | HQ012081 | MA098 | Mexican American | Central America | Present Study |
| 75 | A2 | HQ012082 | MA099 | Mexican American | Central America | Present Study |
| 76 | A2 | HQ012083 | MA100 | Mexican American | Central America | Present Study |
| 77 | A2 | HQ012084 | MA102 | Mexican American | Central America | Present Study |
| 78 | A2 | HQ012085 | MA103 | Mexican American | Central America | Present Study |
| 79 | A2 | HQ012086 | MA108 | Mexican American | Central America | Present Study |
| 80 | A2 | HQ012087 | MA111 | Mexican American | Central America | Present Study |
| 81 | A2 | HQ012088 | MA115 | Mexican American | Central America | Present Study |
| 82 | A2 | HQ012089 | MA116 | Mexican American | Central America | Present Study |
| 83 | A2 | HQ012090 | MA117 | Mexican American | Central America | Present Study |
| 84 | A2 | HQ012091 | MA118 | Mexican American | Central America | Present Study |
| 85 | A2 | HQ012092 | MA119 | Mexican American | Central America | Present Study |
| 86 | A2 | HQ012093 | MA120 | Mexican American | Central America | Present Study |
| 87 | A2 | HQ012094 | MA124 | Mexican American | Central America | Present Study |
| 88 | A2 | HQ012095 | MA125 | Mexican American | Central America | Present Study |
| 89 | A2 | HQ012096 | MA129 | Mexican American | Central America | Present Study |
| 90 | A2 | HQ012097 | MA130 | Mexican American | Central America | Present Study |
| 91 | A2 | HQ012098 | MA132 | Mexican American | Central America | Present Study |
| 92 | A2 | HQ012099 | MA134 | Mexican American | Central America | Present Study |
| 93 | A2 | HQ012100 | MA136 | Mexican American | Central America | Present Study |
| 94 | A2 | HQ012101 | MA137 | Mexican American | Central America | Present Study |
| 95 | A2 | HQ012102 | MA140 | Mexican American | Central America | Present Study |
| 96 | A2 | HQ012103 | MA143 | Mexican American | Central America | Present Study |
| 97 | A2 | HQ012104 | MA145 | Mexican American | Central America | Present Study |
| 98 | A2 | HQ012105 | MA146 | Mexican American | Central America | Present Study |
| 99 | A2 | HQ012106 | MA148 | Mexican American | Central America | Present Study |
| 100 | A2 | HQ012107 | MA152 | Mexican American | Central America | Present Study |
| 101 | A2 | HQ012108 | MA155 | Mexican American | Central America | Present Study |
| 102 | A2 | HQ012109 | MA156 | Mexican American | Central America | Present Study |
| 103 | A2 | HQ012110 | MA157 | Mexican American | Central America | Present Study |
| 104 | A2 | HQ012111 | MA159 | Mexican American | Central America | Present Study |
| 105 | A2 | HQ012112 | MA161 | Mexican American | Central America | Present Study |
| 106 | A2 | HQ012113 | MA164 | Mexican American | Central America | Present Study |
| 107 | A2 | HQ012114 | MA166 | Mexican American | Central America | Present Study |
| 108 | A2 | HQ012115 | MA167 | Mexican American | Central America | Present Study |
| 109 | A2 | HQ012116 | MA168 | Mexican American | Central America | Present Study |
| 110 | A2 | HQ012117 | MA171 | Mexican American | Central America | Present Study |
| 111 | A2 | HQ012118 | MA172 | Mexican American | Central America | Present Study |
| 112 | A2 | HQ012119 | MA173 | Mexican American | Central America | Present Study |
| 113 | A2 | HQ012120 | MA176 | Mexican American | Central America | Present Study |
| 114 | A2 | HQ012121 | MA178 | Mexican American | Central America | Present Study |
| 115 | A2 | HQ012122 | MA190 | Mexican American | Central America | Present Study |
| 116 | A2 | HQ012123 | MA193 | Mexican American | Central America | Present Study |
| 117 | A2 | HQ012124 | MA196 | Mexican American | Central America | Present Study |
| 118 | A2 | HQ012125 | MA197 | Mexican American | Central America | Present Study |
| 119 | A2 | HQ012126 | MA199 | Mexican American | Central America | Present Study |
| 120 | A2 | HQ012127 | MA201 | Mexican American | Central America | Present Study |
| 121 | A2 | HQ012128 | MA202 | Mexican American | Central America | Present Study |
| 122 | A2 | HQ012129 | MA206 | Mexican American | Central America | Present Study |
| 123 | A2 | HQ012130 | MA207 | Mexican American | Central America | Present Study |
| 124 | A2 | HQ012131 | MA209 | Mexican American | Central America | Present Study |
| 125 | A2 | HQ012132 | MA210 | Mexican American | Central America | Present Study |
| 126 | A2 | HQ012133 | MA211 | Mexican American | Central America | Present Study |
| 127 | A2 | EU095194 | ACHE30 | Ache | South America | Fagundes et al., 2008 |
| 128 | A2 | EU095528 | Arsario20 | Arsario | South America | Tamm et al., 2007 |
| 129 | A2 | EU095529 | Cayapa511 | Cayapa | South America | Tamm et al., 2007 |
| 130 | A2 | EU095530 | Cayapa522 | Cayapa | South America | Tamm et al., 2007 |
| 131 | A2 | EU095205 | GRC149 | Guarani/Rio-das-Cobras | South America | Fagundes et al., 2008 |
| 132 | A2 | EF657246 | 112 | ** | North America | Herrnstadt et al, 2002 |
| 133 | A2 | EF657255 | 120 | ** | North America | Herrnstadt et al, 2002 |
| 134 | A2 | EF657254 | 12 | ** | North America | Herrnstadt et al, 2002 |
| 135 | A2 | EF657275 | 139 | ** | North America | Herrnstadt et al, 2002 |
| 136 | A2 | EF657310 | 170 | ** | North America | Herrnstadt et al, 2002 |
| 137 | A2 | EF657311 | 171 | ** | North America | Herrnstadt et al, 2002 |
| 138 | A2 | EF657316 | 176 | ** | North America | Herrnstadt et al, 2002 |
| 139 | A2 | EF657325 | 184 | ** | North America | Herrnstadt et al, 2002 |
| 140 | A2 | EF657344 | 200 | ** | North America | Herrnstadt et al, 2002 |
| 141 | A2 | EF657389 | 241 | ** | North America | Herrnstadt et al, 2002 |
| 142 | A2 | EF657392 | 244 | ** | North America | Herrnstadt et al, 2002 |
| 143 | A2 | EF657395 | 247 | ** | North America | Herrnstadt et al, 2002 |
| 144 | A2 | EF657486 | 329 | ** | North America | Herrnstadt et al, 2002 |
| 145 | A2 | EF657488 | 330 | ** | North America | Herrnstadt et al, 2002 |
| 146 | A2 | EF657489 | 331 | ** | North America | Herrnstadt et al, 2002 |
| 147 | A2 | EF657490 | 332 | ** | North America | Herrnstadt et al, 2002 |
| 148 | A2 | EF657536 | 374 | ** | North America | Herrnstadt et al, 2002 |
| 149 | A2 | EF657538 | 376 | ** | North America | Herrnstadt et al, 2002 |
| 150 | A2 | EF657566 | 400 | ** | North America | Herrnstadt et al, 2002 |
| 151 | A2 | EF657578 | 411 | ** | North America | Herrnstadt et al, 2002 |
| 152 | A2 | EF657582 | 415 | ** | North America | Herrnstadt et al, 2002 |
| 153 | A2 | EF657585 | 418 | ** | North America | Herrnstadt et al, 2002 |
| 154 | A2 | EF657608 | 439 | ** | North America | Herrnstadt et al, 2002 |
| 155 | A2 | EF657630 | 459 | ** | North America | Herrnstadt et al, 2002 |
| 156 | A2 | EF657712 | 532 | ** | North America | Herrnstadt et al, 2002 |
| 157 | A2 | EU431081 | IA_C3 | ** | North America | Achilli et al., 2008 |
| 158 | A2 | EU431082 | IA_H4 | ** | North America | Achilli et al., 2008 |
| 159 | A2 | EU095545 | Kogui39 | Kogui | South America | Tamm et al., 2007 |
| 160 | A2 | EU095204 | KTN130 | Katuena | South America | Fagundes et al., 2008 |
| 161 | A2 | AY195786 | Na5a | Native American | ** | Mishmar et al., 2003 |
| 162 | A2 | EU095201 | PTJ03 | Poturujara | South America | Fagundes et al., 2008 |
| 163 | A2 | EU095198 | SURU01 | Surui | South America | Fagundes et al., 2008 |
| 164 | A2 | EF079873 | Tor22 | ** | Central America | Achilli et al., 2008 |
| 165 | A2 | EU095195 | WAI01 | Waiwai | South America | Fagundes et al., 2008 |
| 166 | A2 | EU095196 | WAI25 | Waiwai | South America | Fagundes et al., 2008 |
| 167 | A2 | EU095552 | Wayuu24 | Wayuu | South America | Tamm et al., 2007 |
| 168 | A2 | EU095199 | WPI167 | Waiapi | South America | Fagundes et al., 2008 |
| 169 | A2 | EU095202 | Y623 | Yanomama | South America | Fagundes et al., 2008 |
| 170 | A2 | EU095200 | Y655 | Yanomama | South America | Fagundes et al., 2008 |
| 171 | A2 | EU095197 | ZOR02 | Zoro | South America | Fagundes et al., 2008 |
| 172 | A2 | DQ112832 | AM17 | Native American | South America | Kivisild et al., 2006 |
| 173 | B2 | EU431083 | IA_E2 | ** | North America | Achilli et al., 2008 |
| 174 | B2 | EU095212 | 1876 | Quechua | South America | Fagundes et al., 2008 |
| 175 | B2 | EU095213 | 1880 | Quechua | South America | Fagundes et al., 2008 |
| 176 | B2 | EU095214 | 1881 | Quechua | South America | Fagundes et al., 2008 |
| 177 | B2 | HQ012134 | MA003 | Mexican American | Central America | Present Study |
| 178 | B2 | HQ012135 | MA006 | Mexican American | Central America | Present Study |
| 179 | B2 | HQ012136 | MA013 | Mexican American | Central America | Present Study |
| 180 | B2 | HQ012137 | MA015 | Mexican American | Central America | Present Study |
| 181 | B2 | HQ012138 | MA016 | Mexican American | Central America | Present Study |
| 182 | B2 | HQ012139 | MA020 | Mexican American | Central America | Present Study |
| 183 | B2 | HQ012140 | MA022 | Mexican American | Central America | Present Study |
| 184 | B2 | HQ012141 | MA027 | Mexican American | Central America | Present Study |
| 185 | B2 | HQ012142 | MA029 | Mexican American | Central America | Present Study |
| 186 | B2 | HQ012143 | MA032 | Mexican American | Central America | Present Study |
| 187 | B2 | HQ012144 | MA050 | Mexican American | Central America | Present Study |
| 188 | B2 | HQ012145 | MA051 | Mexican American | Central America | Present Study |
| 189 | B2 | HQ012146 | MA055 | Mexican American | Central America | Present Study |
| 190 | B2 | HQ012147 | MA064 | Mexican American | Central America | Present Study |
| 191 | B2 | HQ012148 | MA071 | Mexican American | Central America | Present Study |
| 192 | B2 | HQ012149 | MA074 | Mexican American | Central America | Present Study |
| 193 | B2 | HQ012150 | MA082 | Mexican American | Central America | Present Study |
| 194 | B2 | HQ012151 | MA085 | Mexican American | Central America | Present Study |
| 195 | B2 | HQ012152 | MA087 | Mexican American | Central America | Present Study |
| 196 | B2 | HQ012153 | MA088 | Mexican American | Central America | Present Study |
| 197 | B2 | HQ012154 | MA089 | Mexican American | Central America | Present Study |
| 198 | B2 | HQ012155 | MA092 | Mexican American | Central America | Present Study |
| 199 | B2 | HQ012156 | MA094 | Mexican American | Central America | Present Study |
| 200 | B2 | HQ012157 | MA101 | Mexican American | Central America | Present Study |
| 201 | B2 | HQ012158 | MA104 | Mexican American | Central America | Present Study |
| 202 | B2 | HQ012159 | MA105 | Mexican American | Central America | Present Study |
| 203 | B2 | HQ012160 | MA109 | Mexican American | Central America | Present Study |
| 204 | B2 | HQ012161 | MA122 | Mexican American | Central America | Present Study |
| 205 | B2 | HQ012162 | MA123 | Mexican American | Central America | Present Study |
| 206 | B2 | HQ012163 | MA126 | Mexican American | Central America | Present Study |
| 207 | B2 | HQ012164 | MA127 | Mexican American | Central America | Present Study |
| 208 | B2 | HQ012165 | MA128 | Mexican American | Central America | Present Study |
| 209 | B2 | HQ012166 | MA133 | Mexican American | Central America | Present Study |
| 210 | B2 | HQ012167 | MA135 | Mexican American | Central America | Present Study |
| 211 | B2 | HQ012168 | MA139 | Mexican American | Central America | Present Study |
| 212 | B2 | HQ012169 | MA141 | Mexican American | Central America | Present Study |
| 213 | B2 | HQ012170 | MA142 | Mexican American | Central America | Present Study |
| 214 | B2 | HQ012171 | MA144 | Mexican American | Central America | Present Study |
| 215 | B2 | HQ012172 | MA153 | Mexican American | Central America | Present Study |
| 216 | B2 | HQ012173 | MA158 | Mexican American | Central America | Present Study |
| 217 | B2 | HQ012174 | MA160 | Mexican American | Central America | Present Study |
| 218 | B2 | HQ012175 | MA162 | Mexican American | Central America | Present Study |
| 219 | B2 | HQ012176 | MA169 | Mexican American | Central America | Present Study |
| 220 | B2 | HQ012177 | MA170 | Mexican American | Central America | Present Study |
| 221 | B2 | HQ012178 | MA175 | Mexican American | Central America | Present Study |
| 222 | B2 | HQ012179 | MA179 | Mexican American | Central America | Present Study |
| 223 | B2 | HQ012180 | MA186 | Mexican American | Central America | Present Study |
| 224 | B2 | HQ012181 | MA187 | Mexican American | Central America | Present Study |
| 225 | B2 | HQ012182 | MA191 | Mexican American | Central America | Present Study |
| 226 | B2 | HQ012183 | MA195 | Mexican American | Central America | Present Study |
| 227 | B2 | HQ012184 | MA208 | Mexican American | Central America | Present Study |
| 228 | B2 | HQ012185 | MA213 | Mexican American | Central America | Present Study |
| 229 | B2 | EU095206 | ACHE78 | Ache | South America | Fagundes et al., 2008 |
| 230 | B2 | EU095532 | Cayapa602 | Cayapa | South America | Tamm et al., 2007 |
| 231 | B2 | EU095535 | Coreguaje1-30 | Coreguaje | South America | Tamm et al., 2007 |
| 232 | B2 | EU095207 | GAVI23 | Gaviao | South America | Fagundes et al., 2008 |
| 233 | B2 | EU095215 | GRC169 | Guarani/Rio-das-Cobras | South America | Fagundes et al., 2008 |
| 234 | B2 | EF657306 | 167 | ** | North America | Herrnstadt et al, 2002 |
| 235 | B2 | EF657319 | 179 | ** | North America | Herrnstadt et al, 2003 |
| 236 | B2 | EF657326 | 185 | ** | North America | Herrnstadt et al, 2004 |
| 237 | B2 | EF657339 | 197 | ** | North America | Herrnstadt et al, 2005 |
| 238 | B2 | EF657340 | 198 | ** | North America | Herrnstadt et al, 2006 |
| 239 | B2 | EF657347 | 203 | ** | North America | Herrnstadt et al, 2007 |
| 240 | B2 | EF657349 | 205 | ** | North America | Herrnstadt et al, 2008 |
| 241 | B2 | EF657413 | 263 | ** | North America | Herrnstadt et al, 2009 |
| 242 | B2 | EF657482 | 325 | ** | North America | Herrnstadt et al, 2010 |
| 243 | B2 | EF657505 | 346 | ** | North America | Herrnstadt et al, 2011 |
| 244 | B2 | EF657530 | 369 | ** | North America | Herrnstadt et al, 2012 |
| 245 | B2 | EF657537 | 375 | ** | North America | Herrnstadt et al, 2013 |
| 246 | B2 | EF657586 | 419 | ** | North America | Herrnstadt et al, 2014 |
| 247 | B2 | EF657617 | 447 | ** | North America | Herrnstadt et al, 2015 |
| 248 | B2 | EU431084 | IA_G1 | ** | North America | Achilli et al., 2008 |
| 249 | B2 | EU095216 | KBK23 | Kayapo-Kubemkokre | South America | Fagundes et al., 2008 |
| 250 | B2 | EU095217 | KBK39 | Kayapo-Kubemkokre | South America | Fagundes et al., 2008 |
| 251 | B2 | EU095218 | KKT01 | Kayapo-Kriketun | South America | Fagundes et al., 2008 |
| 252 | B2 | EU095219 | KRC33 | Guarani/Rio-das-Cobras | South America | Fagundes et al., 2008 |
| 253 | B2 | EU095220 | KTN209 | Katuena | South America | Fagundes et al., 2008 |
| 254 | B2 | AF347001 | Pinman 26/27 | Pima | North America | Ingman et al., , 2000 |
| 255 | B2 | AY195749 | Na1B | Native-American | ** | Mishmar et al., 2003 |
| 256 | B2 | EU095546 | Ngöbe14 | Ngoebe | Central America | Tamm et al., 2007 |
| 257 | B2 | EU095208 | POMO01 | Pomo | North America | Fagundes et al., 2008 |
| 258 | B2 | EF079874 | Tor23 | ** | Central America | Achilli et al., 2008 |
| 259 | B2 | EU095209 | WAI24 | Waiwai | South America | Fagundes et al., 2008 |
| 260 | B2 | EU095548 | Waunana2-8 | Waunana | South America | Tamm et al., 2007 |
| 261 | B2 | EU095551 | Wayuu17 | Wayuu | South America | Tamm et al., 2007 |
| 262 | B2 | EU095550 | Wayuu7 | Wayuu | South America | Tamm et al., 2007 |
| 263 | B2 | EU095210 | XAV04 | Xavante | South America | Fagundes et al., 2008 |
| 264 | B2 | EU095211 | XAV12 | Xavante | South America | Fagundes et al., 2008 |
| 265 | B2 | EU095221 | Y637 | Yanomama | South America | Fagundes et al., 2008 |
| 266 | B2 | DQ112790 | AM15 | Colombinan | South America | Kivisild2006 |
| 267 | B2 | DQ112791 | AM16 | Colombinan | South America | Kivisild2006 |
| 268 | B2 | DQ112889 | AM12 | Mayan | Central America | Kivisild2006 |
| 269 | C1a | AY519496 | Ulchi(AY519496) | Ulchi | Siberia | Starikovskaya et al., 2005 |
| 270 | C1a | EU007858 | 160 | Nanaitci | Siberia | Ingman et al., 2007 |
| 271 | C1a | EF153779 | Buryat324 | Buryat | Siberia | Derenko et al., 2007 |
| 272 | C1a | AP008311 | TC52 | Japanese | Asia | Tanaka et al., 2004 |
| 273 | C1d | HQ012239 | MA079 | Mexican American | Central America | Present Study |
| 274 | C1b | HQ012204 | MA068 | Mexican American | Central America | Present Study |
| 275 | C1b | HQ012212 | MA188 | Mexican American | Central America | Present Study |
| 276 | C1b | HQ012189 | MA024 | Mexican American | Central America | Present Study |
| 277 | C1b | HQ012197 | MA044 | Mexican American | Central America | Present Study |
| 278 | C1b | HQ012191 | MA031 | Mexican American | Central America | Present Study |
| 279 | C1d | HQ012240 | MA096 | Mexican American | Central America | Present Study |
| 280 | C1b | HQ012190 | MA030 | Mexican American | Central America | Present Study |
| 281 | C1b | HQ012196 | MA041 | Mexican American | Central America | Present Study |
| 282 | C1b | HQ012215 | MA203 | Mexican American | Central America | Present Study |
| 283 | C1c | HQ012229 | MA183 | Mexican American | Central America | Present Study |
| 284 | C1c | HQ012225 | MA121 | Mexican American | Central America | Present Study |
| 285 | C1c | HQ012218 | MA002 | Mexican American | Central America | Present Study |
| 286 | C1b | HQ012199 | MA052 | Mexican American | Central America | Present Study |
| 287 | C1b | HQ012200 | MA056 | Mexican American | Central America | Present Study |
| 288 | C1b | HQ012187 | MA011 | Mexican American | Central America | Present Study |
| 289 | C1d | HQ012235 | MA053 | Mexican American | Central America | Present Study |
| 290 | C1c | HQ012230 | MA189 | Mexican American | Central America | Present Study |
| 291 | C1b | HQ012211 | MA185 | Mexican American | Central America | Present Study |
| 292 | C1c | HQ012221 | MA073 | Mexican American | Central America | Present Study |
| 293 | C1c | HQ012219 | MA008 | Mexican American | Central America | Present Study |
| 294 | C1b | HQ012193 | MA036 | Mexican American | Central America | Present Study |
| 295 | C1c | HQ012233 | MA212 | Mexican American | Central America | Present Study |
| 296 | C1c | HQ012231 | MA198 | Mexican American | Central America | Present Study |
| 297 | C1b | HQ012186 | MA004 | Mexican American | Central America | Present Study |
| 298 | C1b | HQ012198 | MA049 | Mexican American | Central America | Present Study |
| 299 | C1b | HQ012194 | MA037 | Mexican American | Central America | Present Study |
| 300 | C1c | HQ012227 | MA174 | Mexican American | Central America | Present Study |
| 301 | C1d | HQ012237 | MA065 | Mexican American | Central America | Present Study |
| 302 | C1b | HQ012208 | MA110 | Mexican American | Central America | Present Study |
| 303 | C1b | HQ012201 | MA058 | Mexican American | Central America | Present Study |
| 304 | C1b | HQ012202 | MA059 | Mexican American | Central America | Present Study |
| 305 | C1b | HQ012192 | MA034 | Mexican American | Central America | Present Study |
| 306 | C1d | HQ012238 | MA072 | Mexican American | Central America | Present Study |
| 307 | C1b | HQ012213 | MA192 | Mexican American | Central America | Present Study |
| 308 | C1b | HQ012210 | MA184 | Mexican American | Central America | Present Study |
| 309 | C1b | HQ012216 | MA205 | Mexican American | Central America | Present Study |
| 310 | C1b | HQ012207 | MA090 | Mexican American | Central America | Present Study |
| 311 | C1c | HQ012232 | MA204 | Mexican American | Central America | Present Study |
| 312 | C1b | HQ012205 | MA076 | Mexican American | Central America | Present Study |
| 313 | C1d | HQ012234 | MA007 | Mexican American | Central America | Present Study |
| 314 | C1d | HQ012244 | MA182 | Mexican American | Central America | Present Study |
| 315 | C1c | HQ012222 | MA107 | Mexican American | Central America | Present Study |
| 316 | C1b | HQ012203 | MA066 | Mexican American | Central America | Present Study |
| 317 | C1b | HQ012209 | MA131 | Mexican American | Central America | Present Study |
| 318 | C1c | HQ012224 | MA113 | Mexican American | Central America | Present Study |
| 319 | C1c | EU431087 | IA_A7 | ** | North America | Achilli et al., 2008 |
| 320 | C1d | HQ012241 | MA147 | Mexican American | Central America | Present Study |
| 321 | C1d | HQ012243 | MA180 | Mexican American | Central America | Present Study |
| 322 | C1c | HQ012223 | MA112 | Mexican American | Central America | Present Study |
| 323 | C1b | HQ012195 | MA040 | Mexican American | Central America | Present Study |
| 324 | C1 | EU095228 | PTJ68 | Poturujara | South America | Fagundes et al., 2008 |
| 325 | C1b | HQ012188 | MA018 | Mexican American | Central America | Present Study |
| 326 | C1c | HQ012220 | MA026 | Mexican American | Central America | Present Study |
| 327 | C1b | HQ012206 | MA078 | Mexican American | Central America | Present Study |
| 328 | C1b | HQ012217 | MA215 | Mexican American | Central America | Present Study |
| 329 | C1c | HQ012228 | MA177 | Mexican American | Central America | Present Study |
| 330 | C1 | EU095226 | 1878 | Quechua | South America | Fagundes et al., 2008 |
| 331 | C1 | EU095231 | Y669 | Yanomama | South America | Fagundes et al., 2008 |
| 332 | C1 | EU095229 | Y591 | Yanomama | South America | Fagundes et al., 2008 |
| 333 | C1 | EU095222 | WAI16 | Waiwai | South America | Fagundes et al., 2008 |
| 334 | C1d | HQ012242 | MA149 | Mexican American | Central America | Present Study |
| 335 | C1c | EU431086 | IA_A3 | ** | North America | Achilli et al., 2008 |
| 336 | C1 | AY195759 | Na4C | Native-American | ** | Mishmar et al., 2003 |
| 337 | C1b4 | EU431085 | IA_F1 | ** | North America | Achilli et al., 2008 |
| 338 | C1b | HQ012214 | MA194 | Mexican American | Central America | Present Study |
| 339 | C1c | HQ012226 | MA165 | Mexican American | Central America | Present Study |
| 340 | C1b | EU095549 | Wayuu4 | Wayuu | South America | Tamm et al., 2007 |
| 341 | C1 | EU095225 | 1875 | Quechua | South America | Fagundes et al., 2008 |
| 342 | C1 | EU095224 | ZOR31 | Zoro | South America | Fagundes et al., 2008 |
| 343 | C1d | HQ012236 | MA057 | Mexican American | Central America | Present Study |
| 344 | C1c | EF079875 | Tor24 | ** | Central America | Achilli et al., 2008 |
| 345 | C1 | EU095223 | ZOR19 | Zoro | South America | Fagundes et al., 2008 |
| 346 | C1 | EU095227 | ARL58 | Arara do Laranjal | South America | Fagundes et al., 2008 |
| 347 | C1c | EU095544 | Kogui12 | Kogui | South America | Tamm et al., 2007 |
| 348 | C1 | EU095230 | Y650 | Yanomama | South America | Fagundes et al., 2008 |
| 349 | C1 | AF347012 | Warao(SPRACI) | Warao | South America | Ingman et al., 2000 |
| 350 | C1c | EU095527 | Arsario5 | Arsario | South America | Tamm et al., 2007 |
| 351 | C1 | AF347013 | Warao(RML) | Warao | South America | Ingman et al., 2000 |
| 352 | C1d | EU095537 | Coreguaje1-54 | Coreguaje | South America | Tamm et al., 2007 |
| 353 | C1d | DQ112789 | AM03 | ** | South America | Kivisild et al., 2006 |
| 354 | C1b | DQ112846 | AM06 | ** | North America | Kivisild et al., 2006 |
| 355 | C1c | DQ112888 | AM04 | ** | Central America | Kivisild et al., 2006 |
| 356 | C1b | EF657584 | 417 | ** | North America | Herrnstadt et al, 2008 |
| 357 | C1d | EF657504 | 345 | ** | North America | Herrnstadt et al, 2008 |
| 358 | C1b | EF657282 | 145 | ** | North America | Herrnstadt et al, 2008 |
| 359 | C1c | EF657324 | 183 | ** | North America | Herrnstadt et al, 2008 |
| 360 | C1c | EF657329 | 188 | ** | North America | Herrnstadt et al, 2008 |
| 361 | C1c | EF657547 | 384 | ** | North America | Herrnstadt et al, 2008 |
| 362 | C1c | EF657588 | 420 | ** | North America | Herrnstadt et al, 2008 |
| 363 | C1d | EF657314 | 174 | ** | North America | Herrnstadt et al, 2008 |
| 364 | C1c | EF657317 | 177 | ** | North America | Herrnstadt et al, 2008 |
| 365 | C1c | EF657355 | 210 | ** | North America | Herrnstadt et al, 2008 |
| 366 | C1d | HM107306 | S-987389 | Tamaulipas | Central America | Perego et al., 2010 |
| 367 | C1d | HM107307 | S-644177 | Guanajuato | Central America | Perego et al., 2010 |
| 368 | C1d | HM107308 | S-683498 | Chihuahua | Central America | Perego et al., 2010 |
| 369 | C1d | HM107309 | SA19 | Kolla – Salta | South America | Perego et al., 2010 |
| 370 | C1d | HM107310 | SA51 | Kolla – Salta | South America | Perego et al., 2010 |
| 371 | C1d2a | HM107311 | Mst42 | Mestizos – Colombia | South America | Perego et al., 2010 |
| 372 | C1d2a | HM107312 | Mst68 | Mestizos – Colombia | South America | Perego et al., 2010 |
| 373 | C1d2a | HM107313 | Mst61 | Mestizos – Colombia | South America | Perego et al., 2010 |
| 374 | C1d2a | HM107314 | Mst50 | Mestizos – Colombia | South America | Perego et al., 2010 |
| 375 | C1d2 | HM107315 | Mst64 | Mestizos – Colombia | South America | Perego et al., 2010 |
| 376 | C1d | HM107316 | ABS174 | Buenos Aires | South America | Perego et al., 2010 |
| 377 | C1d | HM107317 | S-635878 | Boyaca, Colombia | South America | Perego et al., 2010 |
| 378 | C1d1a1 | HM107318 | S-632547 | Oklahoma, USA | North America | Perego et al., 2010 |
| 379 | C1d1a1 | HM107319 | S-914766 | Montana, USA | North America | Perego et al., 2010 |
| 380 | C1d1a1 | HM107320 | S-677163 | Quebec, Canada | North America | Perego et al., 2010 |
| 381 | C1d1a1 | HM107321 | S-678282 | Zacatecas | Central America | Perego et al., 2010 |
| 382 | C1d1a | HM107322 | S-631499 | Sonora | Central America | Perego et al., 2010 |
| 383 | C1d1b | HM107323 | SA27 | Kolla – Salta | South America | Perego et al., 2010 |
| 384 | C1d1b | HM107324 | DI23 | Diaguita – Catamarca | South America | Perego et al., 2010 |
| 385 | C1d1b | HM107325 | SA40 | Kolla – Salta | South America | Perego et al., 2010 |
| 386 | C1d1b1 | HM107326 | DI22 | Diaguita – Catamarca | South America | Perego et al., 2010 |
| 387 | C1d1b1 | HM107327 | ABS228 | Buenos Aires | South America | Perego et al., 2010 |
| 388 | C1d1b1 | HM107328 | ARN116 | Rìo Negro | South America | Perego et al., 2010 |
| 389 | C1d1b1 | HM107329 | ABS299 | Buenos Aires | South America | Perego et al., 2010 |
| 390 | C1d1b1 | HM107330 | ACO388 | Corrientes | South America | Perego et al., 2010 |
| 391 | C1d1b1 | HM107331 | S-629812 | Flores, Uruguay | South America | Perego et al., 2010 |
| 392 | C1d1b1 | HM107332 | ABS284 | Buenos Aires | South America | Perego et al., 2010 |
| 393 | C1d1b1 | HM107333 | SA53 | Kolla – Salta | South America | Perego et al., 2010 |
| 394 | C1d1c | HM107334 | S-934519 | Oaxaca | Central America | Perego et al., 2010 |
| 395 | C1d1c1 | HM107335 | S-658745 | Texas, USA | North America | Perego et al., 2010 |
| 396 | C1d1c1 | HM107336 | S-635687 | Texas, USA | North America | Perego et al., 2010 |
| 397 | C1d1c1 | HM107337 | S-643125 | Michigan, USA | North America | Perego et al., 2010 |
| 398 | C1d1 | HM107338 | S-689881 | Rio Grande do Sul, Brazil | South America | Perego et al., 2010 |
| 399 | C1d1 | HM107339 | S-923879 | Lima, Peru | South America | Perego et al., 2010 |
| 400 | C1d1 | HM107340 | ABS229 | Buenos Aires | South America | Perego et al., 2010 |
| 401 | C1d1 | HM107341 | S-987167 | Loreto, Peru | South America | Perego et al., 2010 |
| 402 | C1d1 | HM107342 | S-919781 | Loreto, Peru | South America | Perego et al., 2010 |
| 403 | C1d1 | HM107343 | S-935165 | Imbabura, Ecuador | South America | Perego et al., 2010 |
| 404 | C1d1 | HM107344 | Mst98 | Mestizos - Colombia | South America | Perego et al., 2010 |
| 405 | C1d1 | HM107345 | Mst43 | Mestizos - Colombia | South America | Perego et al., 2010 |
| 406 | C1d1d | HM107346 | ABS326 | Buenos Aires | South America | Perego et al., 2010 |
| 407 | C1d1d | HM107347 | S-681747 | Rio Grande do Sul, Brazil | South America | Perego et al., 2010 |
| 408 | C1d1d | HM107348 | S-681241 | Uruguay | South America | Perego et al., 2010 |
| 409 | C1d1 | HM107349 | S-649798 | Minas Gerais, Brazil | South America | Perego et al., 2010 |
| 410 | C1d1e | HM107350 | S-686135 | Bio-bío, Chile | South America | Perego et al., 2010 |
| 411 | C1d1e | HM107351 | ARN112 | Rìo Negro | South America | Perego et al., 2010 |
| 412 | C1d1 | HM107352 | S-919735 | Cajamarca, Peru | South America | Perego et al., 2010 |
| 413 | C1d1 | HM107353 | S-984386 | Huanucu, Peru | South America | Perego et al., 2010 |
| 414 | C1d1 | HM107354 | S-657487 | Puca Puca, Peru | South America | Perego et al., 2010 |
| 415 | C1d1 | HM107355 | ABS155 | Buenos Aires | South America | Perego et al., 2010 |
| 416 | C1d1 | HM107356 | S-681199 | Mato Grosso do Sul, Brazil | South America | Perego et al., 2010 |
| 417 | C1d1 | HM107357 | S-932862 | Chaco, Paraguay | South America | Perego et al., 2010 |
| 418 | C1d1 | HM107358 | SA11 | Kolla – Salta | South America | Perego et al., 2010 |
| 419 | C1d1 | HM107359 | S-938246 | Piura, Peru | South America | Perego et al., 2010 |
| 420 | C1d1 | HM107360 | S-939485 | Huancavelica, Peru | South America | Perego et al., 2010 |
| 421 | C1d1 | HM107361 | ACO394 | Corrientes | South America | Perego et al., 2010 |
| 422 | C1d1 | HM107362 | S-686784 | Los Lagos, Chile | South America | Perego et al., 2010 |
| 423 | C1d1 | HM107363 | S-686788 | Los Lagos, Chile | South America | Perego et al., 2010 |
| 424 | C1d1 | HM107364 | S-635448 | Oklahoma, USA | North America | Perego et al., 2010 |
| 425 | C1d1 | HM107365 | S-996796 | Kuna Yala, Panama | Central America | Perego et al., 2010 |
| 426 | C1d1 | HM107366 | S-915151 | Panama | Central America | Perego et al., 2010 |
| 427 | C1d1 | HM107367 | S-915473 | Darien, Panama | Central America | Perego et al., 2010 |
| 428 | C1d1 | HM107368 | S-671578 | Puerto Cabezas, Nicaragua | Central America | Perego et al., 2010 |
| 429 | C4a | EU482322 | Yuk1 | Yukaghir | Siberia | Volodko et al., 2008 |
| 430 | C4a | EU007861 | Yakut165 | Yakut | Siberia | Ingman et al., 2007 |
| 431 | C4a | AF346979 | Evenki44 | EvenkiEn | Siberia | Ingman et al., 2000 |
| 432 | C4a | AY519485 | Evenk(AY519485) | EvenkiEn | Siberia | Starikovskaya et al., 2005 |
| 433 | C4a | EU482361 | EvOh14 | Evenk | Siberia | Volodko et al., 2008 |
| 434 | C4a | EU482323 | Yuk2 | Yukaghir | Siberia | Volodko et al., 2008 |
| 435 | C4a | EU482332 | Yuk39 | Yukaghir | Siberia | Volodko et al., 2008 |
| 436 | C4a | EU482380 | Tub28 | Tubalar | Siberia | Volodko et al., 2008 |
| 437 | C4a | EU482371 | NgnV20 | Nganasan | Siberia | Volodko et al., 2008 |
| 438 | C4a | AY615360 | Tofalar | Tofalar | Siberia | Starikovskaya et al., 2005 |
| 439 | C4a | AY195753 | As6c | EvenkiEn | Siberia | Mishmar et al., 2003 |
| 440 | C4a | AY255174 | XJ8435 | Chinese | Asia | Kong et al., 2003 |
| 441 | C4b | EU482304 | Yuk50 | Yukaghir | Siberia | Volodko et al., 2008 |
| 442 | C4b | EU482310 | Yuk64 | Yukaghir | Siberia | Volodko et al., 2008 |
| 443 | C4b | EU482312 | Yuk69 | Yukaghir | Siberia | Volodko et al., 2008 |
| 444 | C4b | EU482313 | Yuk74 | Yukaghir | Siberia | Volodko et al., 2008 |
| 445 | C4b | EU482314 | Yuk76 | Yukaghir | Siberia | Volodko et al., 2008 |
| 446 | C4b | EU482315 | Yuk79 | Yukaghir | Siberia | Volodko et al., 2008 |
| 447 | C4b | AF346970 | Buriat288 | Buryat | Siberia | Ingman et al., 2000 |
| 448 | C4b | AY195772 | As5c | ** | Siberia | Mishmar et al., 2003 |
| 449 | C4b | AF346991 | Khirgiz31 | Khirgiz | Siberia | Ingman et al., 2000 |
| 450 | C4b | EU482317 | Yuk85 | Yukaghir | Siberia | Volodko et al., 2008 |
| 451 | C4b | EU482368 | NgnA60 | Naganasan-Avam | Siberia | Volodko et al., 2008 |
| 452 | C4b | AY570526 | Tuvli(AY570526) | Tuvli | Siberia | Starikovskaya et al., 2005 |
| 453 | C4b | EU007879 | 200 | Mansi | Siberia | Ingman et al., 2007 |
| 454 | C4b | EU482324 | Yuk3 | Yukaghir | Siberia | Volodko et al., 2008 |
| 455 | C4b | EU482362 | Yuk40 | Yukaghir | Siberia | Volodko et al., 2008 |
| 456 | C4b | AY195763 | As4c | ** | Siberia | Mishmar et al., 2003 |
| 457 | C4b | AY519487 | Koryak(AY519487) | Koryak | Siberia | Starikovskaya et al., 2005 |
| 458 | C4b | EU482375 | Tub7 | Tubalar | Siberia | Volodko et al., 2008 |
| 459 | C4b | AY519490 | Nganasan(AY519490) | NganasanV | Siberia | Starikovskaya et al., 2005 |
| 460 | C4b | EU482370 | NgnV18 | Nganasan-Vadei | Siberia | Volodko et al., 2008 |
| 461 | C4a | EU482326 | Yuk8 | Yukaghir | Siberia | Volodko et al., 2008 |
| 462 | C4a | EU482330 | Yuk24 | Yukaghir | Siberia | Volodko et al., 2008 |
| 463 | C4c | EU095543 | Ijka72 | Ijka | South America | Tamm et al., 2007 |
| 464 | C4c | EU095543 | SHU01 | Suswap Speaker | North America | Malhi et al., 2010 |
| 465 | D4h3a | HQ012263 | MA046 | Mexican American | Central America | Present Study |
| 466 | D1 | HQ012245 | MA021 | Mexican American | Central America | Present Study |
| 467 | D1 | HQ012255 | MA151 | Mexican American | Central America | Present Study |
| 468 | D1 | HQ012246 | MA045 | Mexican American | Central America | Present Study |
| 469 | D1 | HQ012247 | MA061 | Mexican American | Central America | Present Study |
| 470 | D1 | HQ012248 | MA069 | Mexican American | Central America | Present Study |
| 471 | D1 | HQ012249 | MA084 | Mexican American | Central America | Present Study |
| 472 | D1 | HQ012250 | MA097 | Mexican American | Central America | Present Study |
| 473 | D1 | HQ012251 | MA106 | Mexican American | Central America | Present Study |
| 474 | D1 | HQ012252 | MA114 | Mexican American | Central America | Present Study |
| 475 | D1 | HQ012253 | MA138 | Mexican American | Central America | Present Study |
| 476 | D1 | HQ012254 | MA150 | Mexican American | Central America | Present Study |
| 477 | D1 | HQ012256 | MA154 | Mexican American | Central America | Present Study |
| 478 | D1 | HQ012257 | MA163 | Mexican American | Central America | Present Study |
| 479 | D1 | HQ012258 | MA181 | Mexican American | Central America | Present Study |
| 480 | D1 | HQ012259 | MA200 | Mexican American | Central America | Present Study |
| 481 | D1 | HQ012260 | MA214 | Mexican American | Central America | Present Study |
| 482 | D1 | EU095536 | Coreguaje1-31 | Coreguaje | South America | Tamm et al., 2007 |
| 483 | D1 | EU095232 | GAVI12 | Gaviao | South America | Fagundes et al., 2008 |
| 484 | D1 | EU095233 | GAVI26 | Gaviao | South America | Fagundes et al., 2008 |
| 485 | D1 | EF657278 | 141 | ** | North America | Herrnstadt et al, 2008 |
| 486 | D1 | EF657333 | 191 | ** | North America | Herrnstadt et al, 2008 |
| 487 | D1 | EF657346 | 202 | ** | North America | Herrnstadt et al, 2008 |
| 488 | D1 | EF657363 | 218 | ** | North America | Herrnstadt et al, 2008 |
| 489 | D1 | EF657506 | 347 | ** | North America | Herrnstadt et al, 2008 |
| 490 | D1 | EF657535 | 373 | ** | North America | Herrnstadt et al, 2008 |
| 491 | D1 | EF657539 | 377 | ** | North America | Herrnstadt et al, 2008 |
| 492 | D1 | EF657595 | 427 | ** | North America | Herrnstadt et al, 2008 |
| 493 | D1 | EF657607 | 438 | ** | North America | Herrnstadt et al, 2008 |
| 494 | D1 | EU431089 | IA_F2 | ** | North America | Achilli et al., 2008 |
| 495 | D1 | EU431088 | IA_G4 | ** | North America | Achilli et al., 2008 |
| 496 | D1 | EU095238 | KTN18 | Katuena | South America | Fagundes et al., 2008 |
| 497 | D1 | AF346984 | GRC150 | Guarani | South America | Ingman el al.,2000 |
| 498 | D1 | AY195748 | Na2D | Native-American | ** | Mishmar et al., 2003 |
| 499 | D1 | EU095239 | PTJ01 | Poturujara | South America | Fagundes et al., 2008 |
| 500 | D1 | EU095234 | SUR22 | Surui | South America | Fagundes et al., 2008 |
| 501 | D1 | EF079876 | Tor25 | ** | Central America | Achilli et al., 2008 |
| 502 | D1 | EU095240 | TYR04 | Tiryo | South America | Fagundes et al., 2008 |
| 503 | D1 | EU095241 | TYR16 | Tiryo | South America | Fagundes et al., 2008 |
| 504 | D1 | EU095235 | WAI05 | Waiwai | South America | Fagundes et al., 2008 |
| 505 | D1 | EU095236 | ZOR23 | Zoro | South America | Fagundes et al., 2008 |
| 506 | D1 | DQ112843 | AM14 | Guarani | South America | Kivisild et al., 2006 |
| 507 | D1 | DQ112772 | AM01 | Brazilian | South America | Kivisild et al., 2006 |
| 508 | D1 | DQ112776 | AM02 | Brazilian | South America | Kivisild et al., 2006 |
| 509 | D1 | DQ112871 | AM07 | Que Chua | South America | Kivisild et al., 2006 |
| 510 | D1 | DQ112872 | AM08 | Pima (Ind) | North America | Kivisild et al., 2006 |
| 511 | D1 | DQ112773 | AM09 | Brazilian | South America | Kivisild et al., 2006 |
| 512 | D1 | DQ112774 | AM10 | Brazilian | South America | Kivisild et al., 2006 |
| 513 | D1 | DQ112775 | AM11 | Brazilian | South America | Kivisild et al., 2006 |
| 514 | D1 | EU095237 | GRC131 | Guarani/Rio-das-Cobras | South America | Fagundes et al., 2008 |
| 515^#^ | D2 | ** | Eskimo XIV | Eskimo | Siberia | Derbeneva et al., 2002 |
| 516^#^ | D2 | ** | Aleut IX | Aleut | Commander Islands | Derbeneva et al., 2002 |
| 517^#^ | D2 | ** | Aleut I-1 | Aleut | Commander Islands | Derbeneva et al., 2002 |
| 518^#^ | D2 | ** | Aleut I-2 | Aleut | Commander Islands | Derbeneva et al., 2002 |
| 519^#^ | D2 | ** | Aleut I-3 | Aleut | Commander Islands | Derbeneva et al., 2002 |
| 520^#^ | D2 | ** | Aleut I-4 | Aleut | Commander Islands | Derbeneva et al., 2002 |
| 521^#^ | D2 | ** | Aleut I-5 | Aleut | Commander Islands | Derbeneva et al., 2002 |
| 522^#^ | D2 | ** | Aleut I-6 | Aleut | Commander Islands | Derbeneva et al., 2002 |
| 523^#^ | D2 | ** | Aleut I-7 | Aleut | Commander Islands | Derbeneva et al., 2002 |
| 524^#^ | D2 | ** | Aleut I-8 | Aleut | Commander Islands | Derbeneva et al., 2002 |
| 525^#^ | D2 | ** | Aleut I-9 | Aleut | Commander Islands | Derbeneva et al., 2002 |
| 526^#^ | D2 | ** | Aleut I-10 | Aleut | Commander Islands | Derbeneva et al., 2002 |
| 527^#^ | D2 | ** | Aleut I-11 | Aleut | Commander Islands | Derbeneva et al., 2002 |
| 528^#^ | D2 | ** | Aleut I-12 | Aleut | Commander Islands | Derbeneva et al., 2002 |
| 529^#^ | D2 | ** | Aleut I-13 | Aleut | Commander Islands | Derbeneva et al., 2002 |
| 530^#^ | D2 | ** | Aleut II-1 | Aleut | Commander Islands | Derbeneva et al., 2002 |
| 531^#^ | D2 | ** | Aleut II-2 | Aleut | Commander Islands | Derbeneva et al., 2002 |
| 532^#^ | D2 | ** | Aleut II-3 | Aleut | Commander Islands | Derbeneva et al., 2002 |
| 533^#^ | D2 | ** | Aleut II-4 | Aleut | Commander Islands | Derbeneva et al., 2002 |
| 534^#^ | D2 | ** | Aleut III-1 | Aleut | Commander Islands | Derbeneva et al., 2002 |
| 535^#^ | D2 | ** | Aleut III-2 | Aleut | Commander Islands | Derbeneva et al., 2002 |
| 536^#^ | D2 | ** | Aleut III-3 | Aleut | Commander Islands | Derbeneva et al., 2002 |
| 537^#^ | D2 | ** | Aleut IV-1 | Aleut | Commander Islands | Derbeneva et al., 2002 |
| 538^#^ | D2 | ** | Aleut IV-2 | Aleut | Commander Islands | Derbeneva et al., 2002 |
| 539^#^ | D2 | ** | Aleut IV-3 | Aleut | Commander Islands | Derbeneva et al., 2002 |
| 540^#^ | D2 | ** | Aleut V | Aleut | Commander Islands | Derbeneva et al., 2002 |
| 541^#^ | D2 | ** | Aleut VI | Aleut | Commander Islands | Derbeneva et al., 2002 |
| 542^#^ | D2 | ** | Aleut VII | Aleut | Commander Islands | Derbeneva et al., 2002 |
| 543^#^ | D2 | ** | Aleut VIII-1 | Aleut | Commander Islands | Derbeneva et al., 2002 |
| 544^#^ | D2 | ** | Aleut VIII-2 | Aleut | Commander Islands | Derbeneva et al., 2002 |
| 545^#^ | D2 | ** | Aleut VIII-3 | Aleut | Commander Islands | Derbeneva et al., 2002 |
| 546^#^ | D2 | ** | Eskimo X | Eskimo | Siberia | Derbeneva et al., 2002 |
| 547^#^ | D2 | ** | Eskimo XI | Eskimo | Siberia | Derbeneva et al., 2002 |
| 548^#^ | D2 | ** | Eskimo XII-1 | Eskimo | Siberia | Derbeneva et al., 2002 |
| 549^#^ | D2 | ** | Eskimo XII-2 | Eskimo | Siberia | Derbeneva et al., 2002 |
| 550^#^ | D2 | ** | Eskimo XII-3 | Eskimo | Siberia | Derbeneva et al., 2002 |
| 551^#^ | D2 | ** | Eskimo XIII | Eskimo | Siberia | Derbeneva et al., 2002 |
| 552 | D2 | EU482386 | Yuk93 | Yukaghir | Siberia | Volodko et al., 2008 |
| 553^#^ | D2 | EU482334 | Ale32 | Tlingit | Commander Island | Volodko et al., 2008 |
| 554*^#^* | D2 | EU482339 | Chuk30 | Chukchi | Siberia | Volodko et al., 2008 |
| 555^#^ | D2 | EU482340 | Chuk36 | Chukchi | Siberia | Volodko et al., 2008 |
| 556^#^ | D2 | EU482357 | Esk43 | Eskimo | Siberia | Volodko et al., 2008 |
| 557 | D2a | EF153788 | Br412 | Buryat | Siberia | Derenko et al., 2007 |
| 558 | D2a | EF153796 | Br608 | Buryat | Siberia | Derenko et al., 2007 |
| 559^#^ | D2b | EF153804 | Chukchi2 | Chukchi | Siberia | Derenko et al., 2007 |
| 560 | D2a | EF153815 | Khm27 | Khamnigan | Siberia | Derenko et al., 2007 |
| 561 | D2a | EF153831 | Yak44 | Yakut | Siberia | Derenko et al., 2007 |
| 562^#^ | D2a | EU095539 | esk2 | Eskimo | Siberia | Tamm et al., 2007 |
| 563 | D2b | EU095541 | Evenk2 | Evenk | Siberia | Tamm et al., 2007 |
| 564 | D2b | EU095542 | Evenk34 | Evenk | Siberia | Tamm et al., 2007 |
| 565^#^ | D2a | EU095534 | chc170 | Chukchi | Siberia | Tamm et al., 2007 |
| 566 | D4e1 | HQ012261 | MA035 | Mexican American | Central America | Present Study |
| 567 | D4e1 | HQ012262 | MA081 | Mexican American | Central America | Present Study |
| 568 | D4e1 | AP008624 | ON138 | Japanese | Asia | Tanaka et al., 2004 |

**Not known

^#^ The samples (n=66) were used as Beringian in the Bayesian skyline and diversity analysis.

The published sequences Herrnstadt et al., 2002 and Kivisild et al., 2006 lacks control region information.
