# Supplementary material for: Large scale mitochondrial sequencing in Mexican Americans suggests a reappraisal of Native American origins
Source: BMC Evol Biol. 2011 Oct 7;11:293. doi: 10.1186/1471-2148-11-293 (PMC3217880; doi:10.1186/1471-2148-11-293)
Supplement: Additional file 3 — Figure Legends for additional file 2. The file contains details on phylogenetic reconstruction, sequences and mtDNA mutation rates used for coalescent age estimates in additional file 2. [file 1471-2148-11-293-S3.DOC]

Additional file 3.

**Figure Legends for Additional file 2 (panels A-D):** **Phylogenetic reconstruction of 568 complete mitochondrial DNA (mtDNA) sequences (Maximum parsimony tree), belonging to Native American haplogroups and Siberian-Asian sister clades is presented in four panels (A-D).** Panel ‘A’- (n=172) Native American haplogroup A2 and Siberian-Asian sister clades A2a, A2b, A4a, A4b and A4c. Panel ‘B’- (n=96) Native American haplogroup B2. Panel ‘C’- (n=196) Native American haplogroup C1 and C4c and Siberian-Asian sister clades C1a, C4a, and C4b. Panel‘D’- (n=104) Native American haplogroup D1, D4e1c and D4h3a and Siberian-Asian sister clades D2 and D4e1a. 215 samples marked as “Present Study” are newly sequenced genomes from our Mexican American samples. 353 other sequences are taken from published sources [23-24, 38, 58, 60-62, 64-65, 72-76, 85]. The published sequences [75-76] lacks control region information and control region information not incorporated in the tree for [24], hence such sequences are place at their tentative best using coding region information. The nucleotide substitutions are listed relative to the revised Cambridge reference sequence [88]. Suffixes A, C, G, and T indicate transversions, "d" signifies a deletion and a plus sign (+) an insertion; recurrent mutations are underlined. The prefix "@" indicates back mutation. The A/C stretch length polymorphism in regions 16180–16193 and 303–315, 522–523 and mutation 16519, all known to be hypervariable, were disregarded for tree reconstruction. The geographical locations of the samples (when known) are identified with colors. For naming the new clades we followed van Oven’s mtDNA tree Build 8 [57] and tried to resolve arising inconsistencies in the light of new data. Coalescent age in thousand years (ky) calculated using on Rho (ρ) statistics [90] and two mutation rates i.e. slow mutation rate of Mishmar et al. [58] and caliberated mutation rates of Soares et al., [59] are indicated in blue and red color respectively. The founder age wherever calculated are italicized.
